# A new green approach for *Lavandula stoechas* aroma recovery and stabilization coupling supercritical CO_2_ and natural deep eutectic solvents

**DOI:** 10.1038/s41598-023-39516-5

**Published:** 2023-08-01

**Authors:** Jelena Vladić, Strahinja Kovačević, Silvia Rebocho, Alexandre Paiva, Stela Jokić, Ana Rita Duarte, Igor Jerković

**Affiliations:** 1 Faculdade de Ciências e Tecnologia, Universidade Nova de Lisboa, 2829-516 Caparica, Portugal; 2 Faculty of Technology, University of Novi Sad, Novi Sad, 21000 Serbia; 3 Faculty of Food Technology Osijek, University of Josip Juraj Strossmayer of Osijek, 31000 Osijek, Croatia; 4 Faculty of Chemistry and Technology, University of Split, 21000 Split, Croatia

**Keywords:** Chemical engineering, Chemical engineering, Green chemistry

## Abstract

This work investigated a green approach to obtain and stabilize *Lavandula stoechas* L. volatile organic compounds with sensory aroma characteristics by using alternative solvents, namely supercritical carbon dioxide (scCO_2_) and deep eutectic solvents (DES). The CO_2_ extracts were dispersed in different DES mixtures (betaine:ethylene glycol (1:3), betaine:glycerol (1:2), and glycerol:glucose (4:1)) and their stability was monitored during 6 months of storage at room temperature by monitoring the headspace (HS) profile. The CO_2_ extract was used as the control. It was initially determined that there was a dominant presence of oxygenated monoterpenes (67.33–77.50%) in the extracts. During storage, significant changes occurred in the samples’ HS, such as the decrease in terpene hydrocarbons which also affected the presence of oxygenated terpenes, which increased in certain cases. Moreover, the highest formation of new components was recorded in the control which could be an indicator of decreased stability. The DESs-CO_2_ were more stable than the CO_2_ control and among them, betaine:ethylene glycol stood out as the most adequate systems for maintaining the stability of *L. stoechas* HS components. For the visual estimation of similarities and dissimilarities among the samples, chemometric pattern recognition approaches were applied including the hierarchical cluster analysis, principal component analysis, and sum of ranking differences.

## Introduction

Various volatile organic compounds (VOCs) are characterized by sensory aroma properties, and they show a variety of odours and are otherwise known as odorants, fragrances, or flavours. These VOCs are used in food, beverages, chemical, cosmetic, perfume, and pharmaceutical industries^1–3^ and the market demand for aroma compounds is in constant growth. According to the Grand View Research^4^, the size of the flavours and fragrances market on the global level was estimated at USD 23.35 billion in 2021 and is expected to increase at compound annual growth rate of 4.3% in the period between 2022 and 2030. Also, there is the immense need and aspiration of the industries to substitute synthetic aromas with natural ones to maintain their competitiveness on the market, which is conditioned by the consumer demand for natural products and regulations. Additionally, considering that the organoleptic experience of the product is one of the key factors determining its acceptance and use, aroma compounds have an important role in the economy^5^. Apart from being used for regulating sensory characteristics of products, aroma volatile compounds possess additional medicinally relevant activities. Therefore, they represent biologically active and important compounds.

Furthermore, the rising demand for volatile components of natural origin create the need for research that aims to establish more efficient, cheaper, simpler, and green solutions for their attainment, as well as identifying new renewable sources of natural aroma volatile compounds.

Conventional methods for obtaining natural flavours and fragrances such as hydro- and steam distillation, maceration, solvent extraction, and *enfleurage* represent processes with numerous disadvantages such as the need for purification, ineffective material utilization, degradation of components, and time-consuming processes^3^. To overcome these shortcomings, supercritical carbon dioxide (CO_2_) extraction was developed and its superiority over conventional methods was demonstrated^6^. Supercritical CO_2_ extraction unifies several beneficial aspects and important preconditions for industrial implementation and remaining competitive on the market: (i) the environment—a more efficient utilization of natural raw materials is achieved and an environmentally-safe solvent is used; (ii) health—clean products without the presence of toxic organic solvents are obtained; (iii) profit—by optimizing processes, efficient utilization and maximal yield are achieved with the minimization of process expenses; the process conditions can be manipulated and varied according to the purpose, and fractioning can be conducted which can increase the utilization of the plant material^7,8^. Furthermore, supercritical CO_2_ extraction lowers the possibility of degradation which can often occur, for instance, during steam distillation^9^. Therefore, the smell of CO_2_ extracts is more similar to the one of the herbal material being extracted. In addition, the stability and longer shelf life of CO_2_ extracts compared to essential oil can be the result of the co-extraction of lipid components of higher molecular weight which do not participate in the formation of the smell but can have a role in the deceleration and/or prevention of the evaporation of aromatic VOCs^3^.

Aroma VOCs are characterized by their tendency towards rapid evaporation, instability, and susceptibility to degradation, which can be induced by different factors such as exposure to oxygen, light, moisture, and similar, during processing, storage, or use^9^. Consequently, the changes in chemical composition, organoleptic properties, and bioactivity can occur. Hence, the instability of aroma components limits their application significantly^2^.

Therefore, apart from the development of the CO_2_ extraction which provides extracts of superior quality compared to the products obtained in a conventional manner, it is necessary to establish the procedures which would provide product stability and, in that way, secure the rational application of the extracts and their extended shelf life. One of the solutions is encapsulation, however, the methods of encapsulation represent complex and expensive procedures of aroma preservation^10^. For these reasons, there is the constant aspiration and need for the improvement and identification of new and simple solutions that would support the growing trend of application and preservation of aroma.

Deep eutectic solvents (DES) represent next-generation alternative solvents where by mixing two or more components in an adequate molar ratio, a liquid system is formed. Due to the formation of hydrogen-bond interactions between components, there is a depression of the melting point of the DES mixture, compared to the individual components. When the DES is composed of components of natural origin such as amino acids, organic acids, alcohols, or sugars, it is defined as a natural deep eutectic solvent (NADES)^11^. They can be used as solvents for extractions and synthesis, chromatographic and biomedical processes^12^. The increasing application of DES is attributed to low melting point, simple and easy preparation, tunability and versatility, and low price. Moreover, considering that DES components are of natural origin, they can be biosynthesized or metabolised and, therefore, DES are considered biocompatible and biodegradable. Together with low toxicity, all these properties make DES environmentally safe solvents^12^. Additionally, DES are characterized by their high thermal stability. Several studies indicated that DES can have the role of stabilizers of different types of components such as pigments^13^, phenolic compounds^14,15^, and enzymes^16,17^. Namely, their solubilizing and stabilizing properties are attributed to the potential formation of the intermolecular interaction with target components^12^.

Coupling the application of supercritical CO_2_ to provide a quality product with the stabilization of the product in DES represents a new approach that can provide a rational utilization of aroma VOCs and create a basis for further development of similar procedures, which can contribute towards new solutions for green aroma stabilisation. For this purpose, *L. stoechas* was selected as the source of VOCs. This herbal species belongs to Lamiaceae family and genus *Lavandula*, it is present across the world and used extensively in traditional medicine^18^.

Therefore, for the first time, this study investigated the integration of the application of green solvents, supercritical CO_2_ and DES, for (i) efficient utilization of *L. stoechas* material and attainment of quality products, and (ii) preservation of the sensory headspace (HS) characteristics of the obtained products.

## Results and discussion

### Stability of ***L. stoechas*** CO_2_ extracts in DES

Among the major environmental factors that can lead to the changes in the quality of lipophilic components and products are temperature, light, and oxygen^19^. Previous studies had reported that during storage of extracts rich in volatile compounds and essential oil at low temperatures of 4 °C and − 20 °C, there are slight changes, while very significant changes occur in the chemical profile of the product during storage at room temperature^20^. DESs are characterized by thermal stability, hence, room temperature for the storage of samples was selected and the HS profile of samples stored at room temperature in transparent containers was monitored.

**Figure 1:**
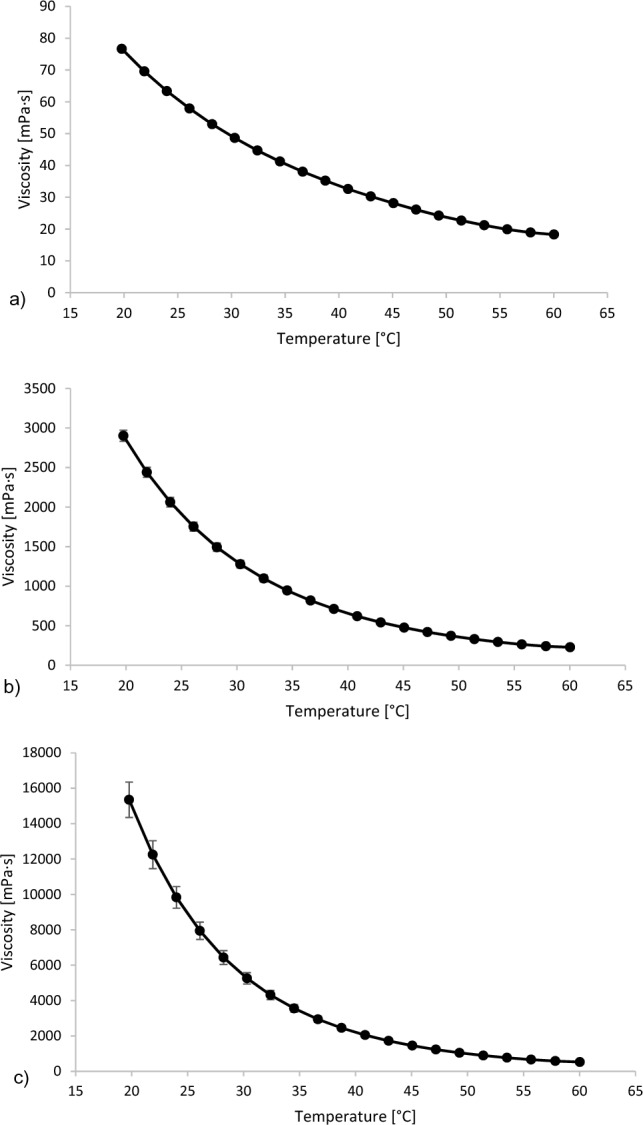
Viscosity of applied DES (**a**) betaine:ethylene glycol (Bet:EG) (1:3); (**b**) betaine:glycerol (Bet:Gly) (1:2), and (**c**) glycerol:glucose (Gly:Glu) (4:1). The figures were created with Microsoft Excel, version 2305. https://www.microsoft.com/en-us/microsoft-365/excel.

The components selected for DESs were betaine, ethylene glycol, glycerol, and glucose. Betaine is obtained as a by-product of sugar products, therefore, it represents a renewable, non-toxic, and biodegradable resource. In addition, betaine has an important function in the cells of most living organisms^21^. For glycerol-based DESs, their adequacy for the separation of terpenes and terpenoids was determined^22^. Furthermore, glycerol also represents a by-product of different production processes, making it highly available. Since the viscosity of DES is one of the parameters that can significantly impact the adequacy of application of DES, different combinations were tested. Namely, to obtain a DES of lower viscosity, ethylene glycol was used, and to obtain a DES of higher viscosity, glucose was selected (Fig. 1).

In our previous study^23^ based on which the conditions for the supercritical CO_2_ extraction were selected, it was determined that by using 200 bar, 40 °C, 20 g/min, and 3 h the total extraction yield was 1.99% (w/w). These results were in accordance with the results reported in Marongiu et al.^24^ where the yield was 1.2–1.8% (w/w) (90 bar and 40 °C). Additionally, the yield of *L. stoechas* essential oil isolated by steam distillation was 0.3–1% (w/w)^25^, while in Gören et al. the achieved yield by hydrodistillation was 1.33% (w/w)^26^.

After dispersing supercritical CO_2_ extracts in different DESs (betaine:ethylen glycol, betaine:glycerol, and glucose: glycerol), the HS profile of the samples was monitored for 6 months (start, 3 months, and 6 months). In the same way, the profile of the control extract (the CO_2_ extract which was not dispersed in DES) was monitored. Detailed and complementary HS profiles of the samples were determined using two fibers of different polarity: divinylbenzene/carboxene/polydimethylsiloxane (DVB/CAR/PDMS) and polydimethylsiloxane/divinylbenzene (PDMS/DVB).

**Figure 2:**
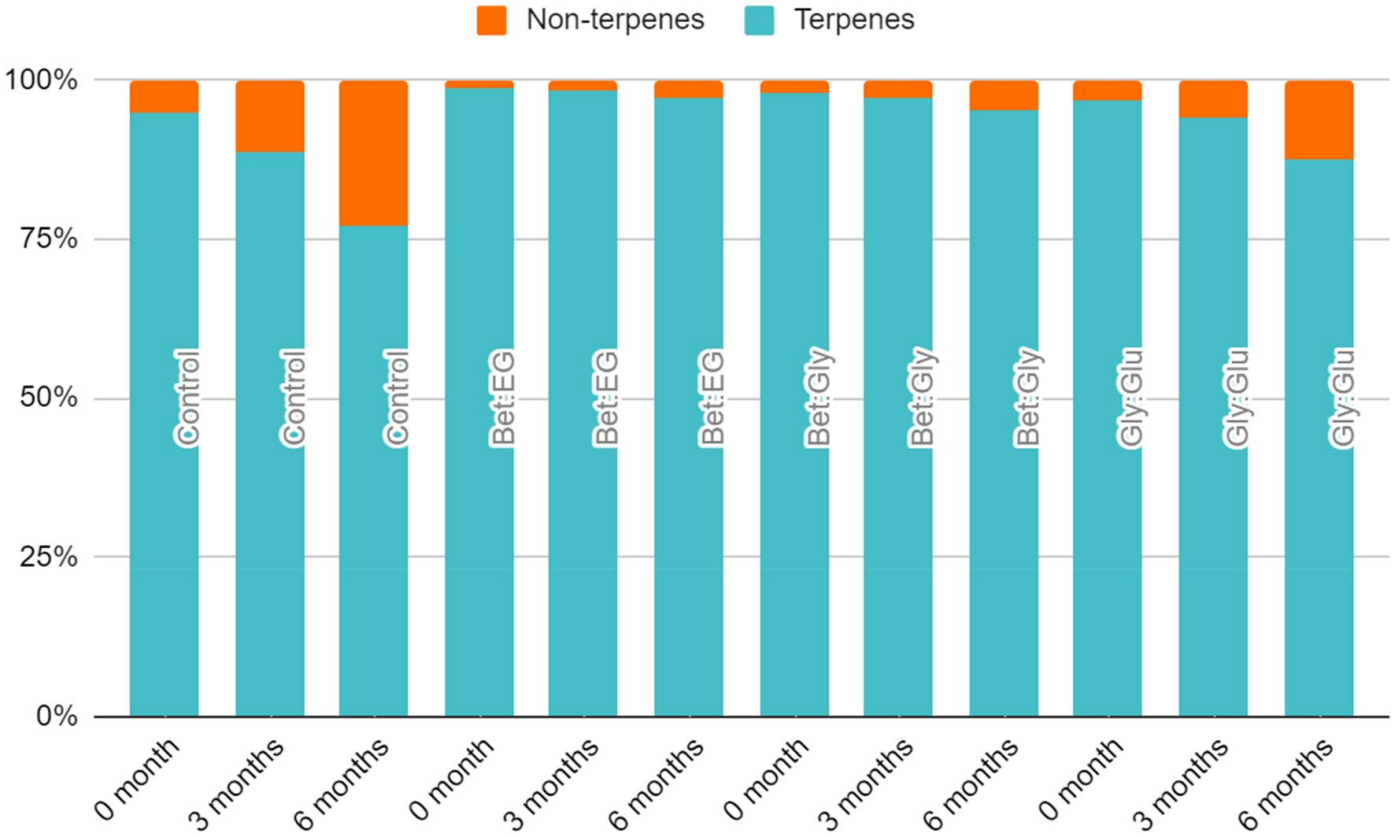
Percentage distribution of terpene and non-terpene compounds in *L. stoechas* extracts (control and DESs-CO_2_ samples) after 0, 3, and 6 months of storage (PDMS/DVB fiber). The figure was created with Microsoft Excel, version 2305. https://www.microsoft.com/en-us/microsoft-365/excel.

A total of 71 headspace components were identified in the samples and they belonged to different classes of organic compounds such as alcohols, hydrocarbons, aldehydes, ketones, acids, esters, and so on (Tables 1 and 2). Moreover, they could be classified into two major groups, terpenes, and non-terpenes (Fig. 2). Terpenes were present in the ranges 76.33–94.21% (DVB/CAR/PDMS) and 73.27–92.55% (PDMS/DVB). The identified terpene components could further be divided into mono- and sesquiterpenes with oxygen and mono- and sesquiterpene hydrocarbons*.* Among the non-terpene compounds (0.92–15.87% DVB/CAR/PDMS and 1.18–21.89% PDMS/DVB), the identified components belonged to the classes of norisoprenoids, organic acids, hydrocarbons, esters, furan and benzene derivatives, and so on (Fig. 2).

**Table 1:** The volatile headspace compounds (%) of *Lavandula stoechas* extracted by headspace solid-phase microextraction (HS-SPME), and analysed by gas chromatography–mass spectrometry (GC–MS) using DVB/CAR/PDMS fiber.

Compound	RI	Control			Bet:EG			Bet:Gly			Gly:Glu		
		Start	3 months	6 months	Start	3 months	6 months	Start	3 months	6 months	Start	3 months	6 months
Monoterpene hydrocarbons													
α-Pinene	945	0.00 ± 0.00^b^	0.09 ± 0.16^b^	0.00 ± 0.00^b^	0.53 ± 0.20^a^	0.07 ± 0.12^b^	0.00 ± 0.00^b^	0.17 ± 0.06^b^	0.00 ± 0.00^b^	0.00 ± 0.00^b^	0.00 ± 0.00^b^	0.00 ± 0.00^b^	0.00 ± 0.00^b^
Camphene	960	0.00 ± 0.00^d^	0.00 ± 0.00^d^	0.04 ± 0.08^ cd^	0.88 ± 0.25^a^	0.48 ± 0.07^b^	0.00 ± 0.00^d^	0.30 ± 0.09^bc^	0.12 ± 0.10^ cd^	0.00 ± 0.00^d^	0.00 ± 0.00^d^	0.00 ± 0.00^d^	0.00 ± 0.00^d^
* p*-Cymene	1032	0.14 ± 0.03^bc^	0.08 ± 0.08^bc^	0.00 ± 0.00^c^	0.33 ± 0.13^a^	0.02 ± 0.02^bc^	0.00 ± 0.00^c^	0.15 ± 0.03^b^	0.00 ± 0.00^c^	0.00 ± 0.00^c^	0.00 ± 0.00^bc^	0.00 ± 0.00^bc^	0.00 ± 0.00^bc^
Limonene	1037	0.05 ± 0.05^ab^	0.00 ± 0.00^b^	0.00 ± 0.00^b^	0.13 ± 0.13^a^	0.00 ± 0.00^b^	0.00 ± 0.00^b^	0.11 ± 0.03^ab^	0.00 ± 0.00^b^	0.00 ± 0.00^b^	0.00 ± 0.00^ab^	0.00 ± 0.00^ab^	0.00 ± 0.00^ab^
Oxygenated monoterpenes													
1,8-Cineole	1040	13.89 ± 4.80^ab^	12.88 ± 7.73^ab^	7.05 ± 4.46^ab^	15.47 ± 2.66^a^	15.26 ± 2.10^a^	12.15 ± 0.09^ab^	6.57 ± 1.76^ab^	5.57 ± 0.26^ab^	3.51 ± 0.50^b^	10.64 ± 3.66^ab^	10.84 ± 3.39^ab^	10.66 ± 5.11^ab^
* Trans*-Linalool oxide	1079	1.08 ± 0.17^def^	1.45 ± 0.12^bcd^	1.59 ± 0.18^b^	0.30 ± 0.01 h	0.47 ± 0.03^g^h	0.70 ± 0.11^ fg^	0.90 ± 0.04^ef^	1.19 ± 0.18^cde^	1.52 ± 0.20^bc^	1.03 ± 0.01^def^	1.45 ± 0.16^bcd^	2.10 ± 0.08^a^
Lavender lactone	1048	1.08 ± 0.17^def^	1.45 ± 0.12^bcd^	1.59 ± 0.18^b^	0.30 ± 0.01 h	0.47 ± 0.03^g^h	0.70 ± 0.11^ fg^	0.90 ± 0.04^ef^	1.19 ± 0.18^cde^	1.52 ± 0.20^bc^	1.03 ± 0.01^def^	1.45 ± 0.16^bcd^	2.10 ± 0.08^a^
Linalool	1104	0.45 ± 0.04^ab^	0.15 ± 0.02^ cd^	0.00 ± 0.00^d^	0.15 ± 0.07^ cd^	0.18 ± 0.08^ cd^	0.32 ± 0.04^bc^	0.61 ± 0.07^a^	0.46 ± 0.09^ab^	0.44 ± 0.06^ab^	0.55 ± 0.15^a^	0.54 ± 0.08^a^	0.00 ± 0.00^d^
Fenchone	1094	19.61 ± 8.88^a^	20.17 ± 8.58^a^	16.10 ± 10.33^a^	20.29 ± 0.82^a^	23.97 ± 1.02^a^	24.15 ± 1.68^a^	30.03 ± 3.54^a^	35.27 ± 5.91^a^	33.00 ± 5.59^a^	20.37 ± 7.95^a^	24.30 ± 8.68^a^	27.51 ± 8.16^a^
Fenchol	1120	1.04 ± 0.24^a^	1.04 ± 0.69^a^	0.49 ± 0.85^a^	0.50 ± 0.04^a^	0.62 ± 0.07^a^	0.50 ± 0.08^a^	0.91 ± 0.09^a^	1.12 ± 0.07^a^	1.35 ± 0.08^a^	0.97 ± 0.25^a^	1.42 ± 0.26^a^	1.80 ± 1.20^a^
α-Campholene aldehyde	1133	0.09 ± 0.08^ab^	0.00 ± 0.00^c^	0.00 ± 0.00^c^	0.02 ± 0.01^bc^	0.00 ± 0.00^c^	0.00 ± 0.00^c^	0.13 ± 0.00^a^	0.01 ± 0.01^c^	0.00 ± 0.00^c^	0.04 ± 0.02^bc^	0.00 ± 0.00^c^	0.00 ± 0.00^c^
Camphor	1152	9.77 ± 3.07^bcd^	11.25 ± 4.02^a-d^	10.48 ± 3.50^bcd^	6.90 ± 0.47^d^	8.27 ± 0.51^ cd^	9.73 ± 0.83^bcd^	11.86 ± 0.65^a-d^	14.67 ± 1.91^ab^	16.98 ± 1.00^a^	7.64 ± 1.38^ cd^	10.24 ± 1.12^bcd^	14.20 ± 2.22^abc^
Chrysanthenone	1156	0.55 ± 0.10^ab^	0.52 ± 0.04^ab^	0.40 ± 0.09^ab^	0.23 ± 0.06^ab^	0.05 ± 0.04^b^	0.39 ± 0.29^ab^	0.30 ± 0.02^ab^	0.16 ± 0.09^ab^	0.58 ± 0.09^a^	0.57 ± 0.06^ab^	0.18 ± 0.22^ab^	0.34 ± 0.48^ab^
Pinocarvone	1169	0.11 ± 0.02^ab^	0.19 ± 0.04^a^	0.04 ± 0.07^bc^	0.03 ± 0.01^bc^	0.00 ± 0.00^c^	0.00 ± 0.00^c^	0.08 ± 0.04^bc^	0.00 ± 0.00^c^	0.00 ± 0.00^c^	0.04 ± 0.02^bc^	0.00 ± 0.00^c^	0.00 ± 0.00^c^
Borneol	1173	0.63 ± 0.12^ab^	0.37 ± 0.14^bc^	0.14 ± 0.24^ cd^	0.03 ± 0.02^d^	0.00 ± 0.00^d^	0.00 ± 0.00^d^	0.42 ± 0.09^abc^	0.45 ± 0.04^ab^	0.49 ± 0.07^ab^	0.39 ± 0.01^abc^	0.51 ± 0.07^ab^	0.72 ± 0.08^a^
Eucarvone	1176	0.76 ± 0.17^abc^	1.01 ± 0.04^ab^	1.23 ± 0.45^a^	0.23 ± 0.11^c^	0.30 ± 0.10^c^	1.04 ± 0.48^ab^	0.43 ± 0.05^bc^	0.50 ± 0.06^bc^	0.79 ± 0.17^abc^	0.61 ± 0.00^abc^	0.49 ± 0.13^bc^	0.17 ± 0.01^c^
Terpinen-4-ol	1183	0.35 ± 0.03^b^	0.18 ± 0.31^b^	0.91 ± 0.22^a^	0.00 ± 0.00^b^	0.00 ± 0.00^b^	0.00 ± 0.00^b^	0.19 ± 0.07^b^	0.00 ± 0.00^b^	0.00 ± 0.00^b^	0.09 ± 0.04^b^	0.12 ± 0.17^b^	0.00 ± 0.00^b^
* p*-Cymen-8-ol	1190	0.28 ± 0.05^a^	0.32 ± 0.12^a^	0.05 ± 0.08^bc^	0.00 ± 0.00^c^	0.00 ± 0.00^c^	0.00 ± 0.00^c^	0.22 ± 0.01^abc^	0.23 ± 0.05^ab^	0.16 ± 0.14^abc^	0.17 ± 0.15^abc^	0.00 ± 0.00^bc^	0.00 ± 0.00^bc^
Verbenone	1194	16.79 ± 3.88^ab^	16.26 ± 1.87^ab^	17.16 ± 4.07^ab^	9.72 ± 1.02^bc^	11.91 ± 1.16^abc^	12.82 ± 2.17^abc^	12.95 ± 0.91^abc^	12.63 ± 1.27^abc^	13.34 ± 2.29^ab^	20.74 ± 1.61^a^	15.72 ± 6.27^ab^	4.09 ± 2.88^c^
Myrtenal	1200	0.09 ± 0.08^abc^	0.14 ± 0.01^a^	0.05 ± 0.09^abc^	0.00 ± 0.00^c^	0.00 ± 0.00^c^	0.00 ± 0.00^c^	0.13 ± 0.03^ab^	0.02 ± 0.03^abc^	0.00 ± 0.00^c^	0.04 ± 0.03^abc^	0.00 ± 0.00^bc^	0.00 ± 0.00^bc^
Estragole	1219	0.29 ± 0.09^ab^	0.38 ± 0.19^ab^	0.11 ± 0.18^ab^	0.26 ± 0.18^ab^	0.00 ± 0.00^b^	0.00 ± 0.00^b^	0.56 ± 0.22^a^	0.45 ± 0.19^ab^	0.21 ± 0.21^ab^	0.15 ± 0.18^ab^	0.08 ± 0.08^ab^	0.00 ± 0.00^b^
* cis*-Chrysanthenone	1214	1.15 ± 0.23^a-d^	1.17 ± 0.33^a^-^d^	1.37 ± 0.37^abc^	0.48 ± 0.06^d^	0.64 ± 0.05^ cd^	0.77 ± 0.04^ cd^	1.02 ± 0.09^bcd^	1.12 ± 0.17^a^-^d^	1.55 ± 0.09^ab^	0.83 ± 0.08^bcd^	0.60 ± 0.81^ cd^	1.89 ± 0.24^a^
Fenchyl acetate	1225	0.38 ± 0.11^abc^	0.50 ± 0.20^a^	0.34 ± 0.19^abc^	0.41 ± 0.07^ab^	0.45 ± 0.08^a^	0.19 ± 0.16^abc^	0.50 ± 0.04^a^	0.42 ± 0.03^ab^	0.09 ± 0.15^bc^	0.36 ± 0.11^abc^	0.27 ± 0.07^abc^	0.00 ± 0.00^c^
Pulegone	1246	0.47 ± 0.11^a^	0.31 ± 0.03^ab^	0.30 ± 0.21^ab^	0.04 ± 0.02^ cd^	0.00 ± 0.00^d^	0.00 ± 0.00^d^	0.26 ± 0.02^abc^	0.01 ± 0.01^d^	0.00 ± 0.00^d^	0.17 ± 0.08^bcd^	0.00 ± 0.00^d^	0.00 ± 0.00^d^
Carvone	1250	0.45 ± 0.11^bcd^	0.60 ± 0.21^a^-^d^	0.53 ± 0.18^a^-^d^	0.39 ± 0.05^ cd^	0.47 ± 0.07^bcd^	0.27 ± 0.24^d^	0.98 ± 0.10^a^	0.89 ± 0.15^ab^	0.77 ± 0.09^abc^	0.70 ± 0.33^a^-^d^	0.66 ± 0.18^a^-^d^	0.69 ± 0.11^a-d^
Isopulegyl acetate*	1284	1.49 ± 0.31^abc^	1.77 ± 0.16^abc^	1.37 ± 0.34^abc^	2.21 ± 0.06^ab^	1.72 ± 0.03^abc^	2.34 ± 1.42^a^	1.61 ± 0.21^abc^	0.88 ± 0.17^bc^	0.52 ± 0.06^c^	0.91 ± 0.04^abc^	0.55 ± 0.08^c^	0.63 ± 0.14^bc^
Bornyl acetate	1290	3.76 ± 0.12^ cd^	4.39 ± 0.52^bcd^	3.22 ± 0.02^de^	6.15 ± 0.47^a^	5.19 ± 0.51^ab^	4.31 ± 0.46^bcd^	4.74 ± 0.41^bc^	3.27 ± 0.59^de^	1.99 ± 0.28f.	2.42 ± 0.32^ef^	1.81 ± 0.13f.	1.19 ± 0.32f.
Lavandulyl acetate	1295	2.25 ± 0.40^b^	0.83 ± 0.56^def^	0.37 ± 0.17^ef^	3.51 ± 0.10^a^	2.31 ± 0.18^b^	1.39 ± 0.25^ cd^	1.97 ± 0.30^bc^	0.81 ± 0.34^def^	0.16 ± 0.04f.	1.23 ± 0.16^cde^	0.82 ± 0.01^def^	0.00 ± 0.00f.
Sesquiterpene hydrocarbons													
Cycloisosativene	1370	0.84 ± 0.24^bc^	0.88 ± 0.24^bc^	0.41 ± 0.17^cde^	2.19 ± 0.19^a^	0.55 ± 0.12^bcd^	0.38 ± 0.33^cde^	0.98 ± 0.15^b^	0.29 ± 0.03^de^	0.00 ± 0.00^e^	0.49 ± 0.16^b^-^e^	0.00 ± 0.00^de^	0.00 ± 0.00^de^
Sativene	1393	0.31 ± 0.09^a^	0.23 ± 0.18^ab^	0.00 ± 0.00^b^	0.24 ± 0.16^ab^	0.00 ± 0.00^b^	0.00 ± 0.00^b^	0.12 ± 0.10^ab^	0.00 ± 0.00^b^	0.00 ± 0.00^b^	0.12 ± 0.00^ab^	0.00 ± 0.00^b^	0.00 ± 0.00^b^
γ-Muurolene	1481	0.10 ± 0.03^a^	0.04 ± 0.07^a^	0.00 ± 0.00^a^	0.10 ± 0.10^a^	0.00 ± 0.00^a^	0.00 ± 0.00^a^	0.09 ± 0.05^a^	0.00 ± 0.00^a^	0.00 ± 0.00^a^	0.04 ± 0.03^a^	0.00 ± 0.00^a^	0.00 ± 0.00^a^
Germacrene D	1485	0.00 ± 0.00^a^	0.03 ± 0.05^a^	0.00 ± 0.00^a^	0.15 ± 0.21^a^	0.00 ± 0.00^a^	0.00 ± 0.00^a^	0.12 ± 0.06^a^	0.00 ± 0.00^a^	0.00 ± 0.00^a^	0.07 ± 0.07^a^	0.00 ± 0.00^a^	0.00 ± 0.00^a^
α-Selinene	1498	0.35 ± 0.08^bc^	0.18 ± 0.16^ cd^	0.00 ± 0.00^d^	0.75 ± 0.10^a^	0.00 ± 0.00^d^	0.00 ± 0.00^d^	0.52 ± 0.10^b^	0.32 ± 0.03^bc^	0.00 ± 0.00^d^	0.34 ± 0.02^bc^	0.53 ± 0.00^ab^	0.00 ± 0.00^d^
α-Muurolene	1503	0.17 ± 0.05^a^	0.20 ± 0.10^a^	0.11 ± 0.20^a^	0.24 ± 0.19^a^	0.00 ± 0.00^a^	0.00 ± 0.00^a^	0.15 ± 0.04^a^	0.00 ± 0.00^a^	0.00 ± 0.00^a^	0.06 ± 0.00^a^	0.00 ± 0.00^a^	0.00 ± 0.00^a^
γ-Cadinene	1518	0.45 ± 0.10^bc^	0.42 ± 0.05^bc^	0.30 ± 0.07^ cd^	0.85 ± 0.01^a^	0.03 ± 0.01^e^	0.00 ± 0.00^e^	0.54 ± 0.13^b^	0.06 ± 0.06^e^	0.00 ± 0.00^e^	0.15 ± 0.08^de^	0.12 ± 0.14^de^	0.00 ± 0.00^e^
* cis*-Calamenene	1528	1.42 ± 0.60^bc^	0.95 ± 0.09^bc^	0.83 ± 0.12^bc^	2.89 ± 1.00^a^	1.02 ± 0.18^bc^	1.05 ± 0.16^bc^	1.22 ± 0.13^bc^	0.54 ± 0.03^c^	0.40 ± 0.09^c^	1.96 ± 0.12^ab^	0.78 ± 0.13^bc^	0.52 ± 0.50^c^
α-Calacorene	1548	0.49 ± 0.03^bcd^	0.61 ± 0.15^a^-^d^	0.54 ± 0.39^a^-^d^	0.60 ± 0.12^a^-^d^	0.40 ± 0.28^d^	0.63 ± 0.11^a^-^d^	0.72 ± 0.13^a^-^d^	0.46 ± 0.04^ cd^	0.19 ± 0.33^d^	1.09 ± 0.03^abc^	1.16 ± 0.07^ab^	1.19 ± 0.19^a^
Cadalene	1679	0.94 ± 0.05^c^	1.40 ± 0.29^bc^	1.96 ± 0.55^b^	1.96 ± 0.28^b^	2.07 ± 0.47^b^	2.05 ± 0.28^b^	1.49 ± 0.26^bc^	1.58 ± 0.17^bc^	1.31 ± 0.30^bc^	2.20 ± 0.37^b^	3.59 ± 0.17^a^	2.09 ± 0.54^b^
β-Eudesmene	1490	1.39 ± 0.30^bc^	0.79 ± 0.79^b^-^f^	0.00 ± 0.00f.	2.35 ± 0.32^a^	0.81 ± 0.14^b^-^f^	0.25 ± 0.43^def^	1.45 ± 0.01^ab^	0.50 ± 0.13^c^-^f^	0.00 ± 0.00f.	1.25 ± 0.09^bcd^	1.08 ± 0.17^b-e^	0.00 ± 0.00^ef^
Oxygenated sesquiterpenes													
Palustrol	1572	0.17 ± 0.02^b^	0.29 ± 0.02^ab^	0.38 ± 0.01^ab^	0.23 ± 0.11^ab^	0.13 ± 0.23^b^	0.14 ± 0.25^b^	0.29 ± 0.04^ab^	0.18 ± 0.16^ab^	0.21 ± 0.18^ab^	0.39 ± 0.11^ab^	0.47 ± 0.17^ab^	0.66 ± 0.06^a^
Caryophyllene oxide	1586	0.38 ± 0.04^a-d^	0.56 ± 0.07^abc^	0.51 ± 0.14^a^-^d^	0.59 ± 0.04^abc^	0.77 ± 0.09^ab^	0.48 ± 0.43^a-d^	0.77 ± 0.15^ab^	0.80 ± 0.05^a^	0.75 ± 0.18^ab^	0.23 ± 0.15^bcd^	0.09 ± 0.08^ cd^	0.00 ± 0.00^d^
Viridiflorol	1595	1.61 ± 0.22f.	1.99 ± 0.14^ef^	2.86 ± 0.93^c^-^f^	2.39 ± 0.20^ef^	3.20 ± 0.46^b-e^	4.25 ± 0.22^bc^	2.65 ± 0.14^def^	3.13 ± 0.37^b-e^	4.02 ± 0.31^bcd^	3.29 ± 0.49^b-e^	4.73 ± 1.39^b^	6.90 ± 0.18^a^
Torreyol	1647	0.24 ± 0.03^a^	0.28 ± 0.01^a^	0.24 ± 0.22^a^	0.26 ± 0.10^a^	0.38 ± 0.24^a^	0.00 ± 0.00^a^	0.30 ± 0.04^a^	0.35 ± 0.03^a^	0.25 ± 0.22^a^	0.33 ± 0.04^a^	0.33 ± 0.04^a^	0.34 ± 0.48^a^
α-Cadinol	1660	3.20 ± 0.60^a^	2.73 ± 0.39^a^	3.86 ± 1.97^a^	3.67 ± 1.00^a^	5.21 ± 1.18^a^	6.64 ± 2.19^a^	4.33 ± 1.35^a^	4.69 ± 0.88^a^	5.56 ± 1.79^a^	2.29 ± 0.18^a^	3.35 ± 0.85^a^	5.15 ± 1.03^a^
Hexahydrofarnesyl acetone	1850	0.04 ± 0.05^b^	0.24 ± 0.01^b^	0.52 ± 0.22^ab^	0.13 ± 0.14^b^	0.16 ± 0.28^b^	0.47 ± 0.41^ab^	0.24 ± 0.06^b^	0.53 ± 0.15^ab^	1.06 ± 0.34^a^	0.01 ± 0.01^b^	0.14 ± 0.21^b^	0.00 ± 0.00^b^
Non-terpenes													
2(5H)-Furanone, 5,5-dimethyl-	961	0.55 ± 0.05^a^	0.49 ± 0.12^a^	0.43 ± 0.14^a^	0.00 ± 0.00^b^	0.00 ± 0.00^b^	0.00 ± 0.00^b^	0.00 ± 0.00^b^	0.00 ± 0.00^b^	0.00 ± 0.00^b^	0.00 ± 0.00^b^	0.00 ± 0.00^b^	0.00 ± 0.00^b^
3-Acetyl-2,4-dimethylfuran	1096	0.88 ± 0.12^c^	0.86 ± 0.12^c^	0.56 ± 0.48^c^	0.45 ± 0.06^c^	0.61 ± 0.09^c^	0.82 ± 0.07^c^	0.63 ± 0.09^c^	0.70 ± 0.13^c^	0.98 ± 0.23^bc^	0.97 ± 0.13^bc^	1.55 ± 0.01^ab^	2.06 ± 0.10^a^
Benzaldehyde	971	0.00 ± 0.00^a^	0.04 ± 0.06^a^	0.00 ± 0.00^a^	0.00 ± 0.00^a^	0.00 ± 0.00^a^	0.00 ± 0.00^a^	0.00 ± 0.00^a^	0.00 ± 0.00^a^	0.00 ± 0.00^a^	0.00 ± 0.00^a^	0.00 ± 0.00^a^	0.00 ± 0.00^a^
Benzyl alcohol	1043	0.00 ± 0.00^c^	0.16 ± 0.07^b^	0.27 ± 0.09^a^	0.00 ± 0.00^c^	0.00 ± 0.00^c^	0.00 ± 0.00^c^	0.00 ± 0.00^c^	0.00 ± 0.00^c^	0.00 ± 0.00^c^	0.00 ± 0.00^c^	0.00 ± 0.00^c^	0.00 ± 0.00^c^
4-Methoxy-benzaldehyde	1265	0.00 ± 0.00^b^	0.00 ± 0.00^b^	0.44 ± 0.17^a^	0.00 ± 0.00^b^	0.00 ± 0.00^b^	0.00 ± 0.00^b^	0.00 ± 0.00^b^	0.00 ± 0.00^b^	0.00 ± 0.00^b^	0.00 ± 0.00^b^	0.00 ± 0.00^b^	0.00 ± 0.00^b^
2-Phenylethanol	1125	0.00 ± 0.00^b^	0.00 ± 0.00^b^	0.89 ± 0.92^a^	0.00 ± 0.00^b^	0.00 ± 0.00^b^	0.00 ± 0.00^b^	0.00 ± 0.00^b^	0.00 ± 0.00^b^	0.00 ± 0.00^b^	0.00 ± 0.00^b^	0.00 ± 0.00^b^	0.00 ± 0.00^b^
Ethanol	< 900	0.12 ± 0.03^de^	0.22 ± 0.02^d^	0.45 ± 0.19^c^	0.00 ± 0.00^e^	0.00 ± 0.00^e^	0.00 ± 0.00^e^	0.00 ± 0.00^e^	0.00 ± 0.00^e^	0.68 ± 0.04^b^	0.00 ± 0.00^e^	0.00 ± 0.00^e^	1.61 ± 0.10^a^
Acetic acid	< 900	1.17 ± 0.33^c^	3.79 ± 0.24^b^	10.05 ± 2.42^a^	0.00 ± 0.00^c^	0.00 ± 0.00^c^	0.00 ± 0.00^c^	0.00 ± 0.00^c^	0.00 ± 0.00^c^	0.87 ± 0.57^c^	0.00 ± 0.00^c^	0.00 ± 0.00^c^	3.85 ± 0.09^b^
Propanoic acid	< 900	0.00 ± 0.00^b^	0.07 ± 0.08^b^	0.18 ± 0.07^a^	0.00 ± 0.00^b^	0.00 ± 0.00^b^	0.00 ± 0.00^b^	0.00 ± 0.00^b^	0.00 ± 0.00^b^	0.00 ± 0.00^b^	0.00 ± 0.00^b^	0.00 ± 0.00^b^	0.00 ± 0.00^b^
2-Methypropanoic acid	< 900	0.01 ± 0.02^c^	0.08 ± 0.01^b^	0.17 ± 0.04^a^	0.00 ± 0.00^c^	0.00 ± 0.00^c^	0.00 ± 0.00^c^	0.00 ± 0.00^c^	0.00 ± 0.00^c^	0.00 ± 0.00^c^	0.00 ± 0.00^c^	0.00 ± 0.00^c^	0.00 ± 0.00^c^
Butanoic acid	< 900	0.00 ± 0.00^b^	0.03 ± 0.03^a^	0.00 ± 0.00^b^	0.00 ± 0.00^b^	0.00 ± 0.00^b^	0.00 ± 0.00^b^	0.00 ± 0.00^b^	0.00 ± 0.00^b^	0.00 ± 0.00^b^	0.00 ± 0.00^b^	0.00 ± 0.00^b^	0.00 ± 0.00^b^
2-Methylbutanoic acid	< 900	0.00 ± 0.00^a^	0.05 ± 0.04^a^	0.04 ± 0.04^a^	0.00 ± 0.00^a^	0.00 ± 0.00^a^	0.00 ± 0.00^a^	0.00 ± 0.00^a^	0.00 ± 0.00^a^	0.00 ± 0.00^a^	0.00 ± 0.00^a^	0.00 ± 0.00^a^	0.00 ± 0.00^a^
3-Methylbut-2-enoic acid	< 900	0.00 ± 0.00^a^	0.02 ± 0.03^a^	0.00 ± 0.00^a^	0.00 ± 0.00^a^	0.00 ± 0.00^a^	0.00 ± 0.00^a^	0.00 ± 0.00^a^	0.00 ± 0.00^a^	0.00 ± 0.00^a^	0.00 ± 0.00^a^	0.00 ± 0.00^a^	0.00 ± 0.00^a^
γ-Butyrolactone	925	0.00 ± 0.00^b^	0.15 ± 0.06^a^	0.17 ± 0.04^a^	0.00 ± 0.00^b^	0.00 ± 0.00^b^	0.00 ± 0.00^b^	0.00 ± 0.00^b^	0.00 ± 0.00^b^	0.00 ± 0.00^b^	0.00 ± 0.00^b^	0.00 ± 0.00^b^	0.00 ± 0.00^b^
Hexan-2,5-dione	938	0.00 ± 0.00^b^	0.02 ± 0.03^ab^	0.07 ± 0.06^a^	0.00 ± 0.00^b^	0.00 ± 0.00^b^	0.00 ± 0.00^b^	0.00 ± 0.00^b^	0.00 ± 0.00^b^	0.00 ± 0.00^b^	0.00 ± 0.00^b^	0.00 ± 0.00^b^	0.00 ± 0.00^b^
Hexanoic acid	981	0.00 ± 0.00^b^	0.09 ± 0.08^b^	0.40 ± 0.30^a^	0.00 ± 0.00^b^	0.00 ± 0.00^b^	0.00 ± 0.00^b^	0.00 ± 0.00^b^	0.00 ± 0.00^b^	0.00 ± 0.00^b^	0.00 ± 0.00^b^	0.00 ± 0.00^b^	0.00 ± 0.00^b^
Oct-1-en-3-ol	985	0.13 ± 0.03^a^	0.00 ± 0.00^a^	0.12 ± 0.20^a^	0.00 ± 0.00^a^	0.00 ± 0.00^a^	0.00 ± 0.00^a^	0.00 ± 0.00^a^	0.00 ± 0.00^a^	0.00 ± 0.00^a^	0.00 ± 0.00^a^	0.00 ± 0.00^a^	0.00 ± 0.00^a^
3-Methylbenzaldehyde	1090	0.00 ± 0.00^a^	0.00 ± 0.00^a^	0.13 ± 0.23^a^	0.00 ± 0.00^a^	0.00 ± 0.00^a^	0.00 ± 0.00^a^	0.00 ± 0.00^a^	0.00 ± 0.00^a^	0.00 ± 0.00^a^	0.00 ± 0.00^a^	0.00 ± 0.00^a^	0.00 ± 0.00^a^
6-Methyl-3,5-heptadien-2-one	1109	0.19 ± 0.08^abc^	0.29 ± 0.05^ab^	0.33 ± 0.17^a^	0.00 ± 0.00^c^	0.00 ± 0.00^c^	0.00 ± 0.00^c^	0.07 ± 0.04^bc^	0.02 ± 0.03^c^	0.12 ± 0.11^abc^	0.04 ± 0.02^bc^	0.13 ± 0.18^abc^	0.00 ± 0.00^c^
Oct-1-en-3-yl acetate	1117	0.11 ± 0.05^ab^	0.12 ± 0.03^ab^	0.04 ± 0.06^bc^	0.19 ± 0.04^a^	0.00 ± 0.00^c^	0.00 ± 0.00^c^	0.16 ± 0.05^a^	0.01 ± 0.01^c^	0.00 ± 0.00^c^	0.01 ± 0.01^bc^	0.00 ± 0.00^c^	0.00 ± 0.00^c^
Methylacetophe-none	1189	0.49 ± 0.10^ab^	0.64 ± 0.02^a^	0.41 ± 0.23^ab^	0.00 ± 0.00^c^	0.00 ± 0.00^c^	0.00 ± 0.00^c^	0.30 ± 0.04^b^	0.34 ± 0.02^b^	0.00 ± 0.00^c^	0.24 ± 0.04^bc^	0.37 ± 0.07^b^	0.00 ± 0.00^c^
Heptadecane	1700	0.10 ± 0.06^a^	0.09 ± 0.03^a^	0.00 ± 0.00^a^	0.11 ± 0.08^a^	0.00 ± 0.00^a^	0.00 ± 0.00^a^	0.06 ± 0.01^a^	0.03 ± 0.05^a^	0.00 ± 0.00^a^	0.06 ± 0.07^a^	0.00 ± 0.00^a^	0.00 ± 0.00^a^
α-Isophorone	1086	0.76 ± 0.23^a^-^d^	0.87 ± 0.16^abc^	0.71 ± 0.33^a^-^e^	0.33 ± 0.09^de^	0.31 ± 0.06^e^	0.61 ± 0.07^b^-^e^	0.59 ± 0.08^b^-^e^	0.70 ± 0.07^a^-^e^	0.53 ± 0.07^cde^	0.83 ± 0.08^a^-^d^	1.05 ± 0.16^ab^	1.19 ± 0.12^a^

Means ± standard deviation followed by different letters in the same row differ significantly, based on Tukey’s test at *p* < 0.05.

**Table 2:** The volatile headspace compounds (%) of *Lavandula stoechas* extracted by headspace solid-phase microextraction (HS-SPME), and analysed by gas chromatography–mass spectrometry (GC–MS) using PDMS/DVB fiber.

Compound	RI	Control			Bet:EG			Bet:Gly			Gly:Glu		
		Start	3 months	6 months	Start	3 months	6 months	Start	3 months	6 months	Start	3 months	6 months
Monoterpene hydrocarbons													
α-Pinene	945	0.00 ± 0.00^c^	0.00 ± 0.00^c^	0.04 ± 0.08^bc^	0.30 ± 0.03^a^	0.05 ± 0.08^bc^	0.00 ± 0.00^c^	0.15 ± 0.03^b^	0.00 ± 0.00^c^	0.00 ± 0.00^c^	0.00 ± 0.00^c^	0.00 ± 0.00^c^	0.00 ± 0.00^c^
Camphene	960	0.00 ± 0.00^c^	0.00 ± 0.00^c^	0.08 ± 0.13^bc^	0.62 ± 0.17^a^	0.28 ± 0.07^b^	0.07 ± 0.12^bc^	0.25 ± 0.05^bc^	0.05 ± 0.08^bc^	0.00 ± 0.00^c^	0.00 ± 0.00^c^	0.00 ± 0.00^c^	0.00 ± 0.00^c^
* p*-Cymene	1032	0.11 ± 0.03^b^	0.00 ± 0.00^c^	0.03 ± 0.05^c^	0.21 ± 0.02^a^	0.07 ± 0.06^bc^	0.00 ± 0.00^c^	0.12 ± 0.03^b^	0.00 ± 0.00^c^	0.00 ± 0.00^c^	0.00 ± 0.00^c^	0.00 ± 0.00^c^	0.00 ± 0.00^c^
Limonene	1037	0.03 ± 0.03^a^	0.03 ± 0.05^a^	0.00 ± 0.00^a^	0.10 ± 0.09^a^	0.00 ± 0.00^a^	0.00 ± 0.00^a^	0.10 ± 0.02^a^	0.00 ± 0.00^a^	0.00 ± 0.00^a^	0.00 ± 0.00^a^	0.00 ± 0.00^a^	0.00 ± 0.00^a^
Oxygenated monoterpenes													
1,8-Cineole	1040	10.65 ± 3.83^ab^	8.48 ± 7.61^ab^	9.07 ± 4.60^ab^	13.70 ± 2.24^ab^	16.26 ± 2.31^a^	14.06 ± 0.98^ab^	5.15 ± 1.16^b^	4.93 ± 0.50^b^	3.98 ± 0.61^b^	9.18 ± 3.44^ab^	11.82 ± 2.83^ab^	11.82 ± 6.87^ab^
* Trans*-Linalool oxide	1079	1.25 ± 0.19^bcd^	1.52 ± 0.21^ab^	1.60 ± 0.15^ab^	0.40 ± 0.01f.	0.37 ± 0.05f.	0.71 ± 0.06^e^f	0.97 ± 0.05^de^	1.15 ± 0.11^ cd^	1.43 ± 0.12^abc^	1.40 ± 0.10^abc^	1.67 ± 0.13^a^	1.78 ± 0.03^a^
Lavender lactone	1048	0.38 ± 0.08^ab^	0.43 ± 0.20^a^	0.50 ± 0.09^a^	0.00 ± 0.00^d^	0.00 ± 0.00^d^	0.00 ± 0.00^d^	0.12 ± 0.01^ cd^	0.15 ± 0.03^bcd^	0.11 ± 0.09^ cd^	0.28 ± 0.02^abc^	0.33 ± 0.01^abc^	0.40 ± 0.04^ab^
Linalool	1104	0.49 ± 0.03^bc^	0.16 ± 0.04^e^f	0.00 ± 0.00f.	0.25 ± 0.02^de^	0.31 ± 0.05^cde^	0.30 ± 0.04^cde^	0.72 ± 0.11^a^	0.49 ± 0.13^bc^	0.36 ± 0.05^bcd^	0.75 ± 0.06^a^	0.55 ± 0.10^ab^	0.00 ± 0.00f.
Fenchone	1094	17.79 ± 8.03^bc^	15.38 ± 5.23^c^	17.65 ± 10.25^bc^	20.01 ± 0.89^abc^	26.19 ± 0.91^abc^	26.27 ± 1.06^abc^	24.57 ± 1.56^abc^	37.56 ± 4.22^a^	35.36 ± 5.41^ab^	19.82 ± 6.33^abc^	28.43 ± 13.03^abc^	28.15 ± 5.73^abc^
Fenchol	1120	1.20 ± 0.27^a^	1.30 ± 1.16^a^	0.47 ± 0.82^a^	0.58 ± 0.07^a^	0.54 ± 0.03^a^	0.55 ± 0.14^a^	0.95 ± 0.09^a^	1.19 ± 0.06^a^	1.40 ± 0.08^a^	1.18 ± 0.23^a^	1.54 ± 0.43^a^	1.67 ± 0.77^a^
α-Campholene aldehyde	1133	0.12 ± 0.03^a^	0.00 ± 0.00^b^	0.00 ± 0.00^b^	0.02 ± 0.00^b^	0.00 ± 0.00^b^	0.00 ± 0.00^b^	0.12 ± 0.01^a^	0.05 ± 0.09^ab^	0.00 ± 0.00^b^	0.01 ± 0.00^b^	0.00 ± 0.00^b^	0.00 ± 0.00^b^
Camphor	1152	9.68 ± 3.00^bc^	11.33 ± 3.56^abc^	10.43 ± 3.71^abc^	7.64 ± 0.72^c^	9.17 ± 0.73^bc^	10.48 ± 0.99^abc^	10.29 ± 1.12^abc^	16.42 ± 1.04^ab^	17.53 ± 0.47^a^	7.58 ± 0.85^c^	5.12 ± 7.02^c^	13.56 ± 0.41^abc^
Chrysanthenone	1156	0.62 ± 0.10^a^	0.53 ± 0.02^ab^	0.42 ± 0.08^abc^	0.26 ± 0.07^bcd^	0.22 ± 0.14^bcd^	0.12 ± 0.21^ cd^	0.29 ± 0.03^bcd^	0.12 ± 0.11^ cd^	0.08 ± 0.14^d^	0.67 ± 0.01^a^	0.03 ± 0.01^d^	0.00 ± 0.00^d^
Pinocarvone	1169	0.20 ± 0.02^a^	0.13 ± 0.05^b^	0.02 ± 0.03^c^	0.02 ± 0.01^c^	0.00 ± 0.00^c^	0.00 ± 0.00^c^	0.13 ± 0.03^b^	0.00 ± 0.00^c^	0.00 ± 0.00^c^	0.02 ± 0.00^c^	0.00 ± 0.00^c^	0.00 ± 0.00^c^
Borneol	1173	0.94 ± 0.17^a^	0.52 ± 0.23^bc^	0.14 ± 0.25^cde^	0.03 ± 0.02^de^	0.00 ± 0.00^e^	0.00 ± 0.00^e^	0.53 ± 0.10^bc^	0.42 ± 0.08^bcd^	0.49 ± 0.12^bc^	0.54 ± 0.03^abc^	0.56 ± 0.06^abc^	0.60 ± 0.06^ab^
Eucarvone	1176	0.91 ± 0.18^abc^	1.03 ± 0.07^ab^	1.16 ± 0.48^a^	0.30 ± 0.09^c^	0.37 ± 0.02^bc^	0.49 ± 0.42^bc^	0.46 ± 0.07^bc^	0.48 ± 0.07^bc^	0.75 ± 0.17^abc^	0.71 ± 0.02^abc^	0.47 ± 0.04^bc^	0.25 ± 0.25^c^
Terpinen-4-ol	1183	0.43 ± 0.05^abc^	0.83 ± 0.72^ab^	1.01 ± 0.40^a^	0.00 ± 0.00^c^	0.00 ± 0.00^c^	0.00 ± 0.00^c^	0.21 ± 0.08^bc^	0.00 ± 0.00^c^	0.00 ± 0.00^c^	0.30 ± 0.05^abc^	0.00 ± 0.00^bc^	0.00 ± 0.00^bc^
* p*-Cymen-8-ol	1190	0.31 ± 0.17^ab^	0.47 ± 0.22^a^	0.10 ± 0.17^b^	0.00 ± 0.00^b^	0.00 ± 0.00^b^	0.00 ± 0.00^b^	0.24 ± 0.02^ab^	0.08 ± 0.14^b^	0.00 ± 0.00^b^	0.20 ± 0.05^ab^	0.00 ± 0.00^b^	0.00 ± 0.00^bc^
Verbenone	1194	18.08 ± 3.56^abc^	18.38 ± 1.87^ab^	15.02 ± 3.15^bc^	11.03 ± 0.88^bcd^	10.72 ± 1.44^ cd^	11.41 ± 1.27^bcd^	13.23 ± 1.14^bc^	12.71 ± 2.28^bc^	12.19 ± 2.84^bc^	24.38 ± 4.17^a^	16.38 ± 3.83^abc^	3.58 ± 3.73^d^
Myrtenal	1200	0.22 ± 0.09^a^	0.17 ± 0.04^a^	0.00 ± 0.00^b^	0.00 ± 0.00^b^	0.00 ± 0.00^b^	0.00 ± 0.00^b^	0.17 ± 0.02^a^	0.04 ± 0.07^b^	0.00 ± 0.00^b^	0.01 ± 0.01^b^	0.00 ± 0.00^b^	0.00 ± 0.00^b^
Estragole	1219	0.30 ± 0.16^ab^	0.30 ± 0.09^ab^	0.09 ± 0.16^ab^	0.23 ± 0.08^ab^	0.00 ± 0.00^b^	0.00 ± 0.00^b^	0.40 ± 0.15^a^	0.34 ± 0.16^ab^	0.11 ± 0.20^ab^	0.14 ± 0.11^ab^	0.03 ± 0.01^ab^	0.00 ± 0.00^ab^
* cis*-Chrysanthenone	1214	1.41 ± 0.31^a^	1.39 ± 0.46^a^	1.17 ± 0.30^ab^	0.62 ± 0.07^bc^	0.51 ± 0.05^bc^	0.52 ± 0.45^bc^	1.13 ± 0.11^ab^	1.12 ± 0.11^ab^	1.42 ± 0.05^a^	1.09 ± 0.00^ab^	0.13 ± 0.07^c^	1.67 ± 0.04^a^
Fenchyl acetate	1225	0.58 ± 0.12^a^	0.49 ± 0.29^ab^	0.28 ± 0.12^a-d^	0.41 ± 0.07^a-d^	0.43 ± 0.09^a-d^	0.31 ± 0.28^a-d^	0.47 ± 0.03^abc^	0.28 ± 0.09^a^-^d^	0.00 ± 0.00^d^	0.40 ± 0.10^a^-^d^	0.07 ± 0.02^bcd^	0.00 ± 0.00^ cd^
Pulegone	1246	0.67 ± 0.14^a^	0.37 ± 0.05^b^	0.23 ± 0.19^bcd^	0.10 ± 0.09^ cd^	0.00 ± 0.00^d^	0.00 ± 0.00^d^	0.30 ± 0.05^bc^	0.05 ± 0.09^ cd^	0.00 ± 0.00^d^	0.19 ± 0.10^bcd^	0.00 ± 0.00^d^	0.00 ± 0.00^d^
Carvone	1250	0.51 ± 0.10^abc^	0.58 ± 0.24^abc^	0.41 ± 0.17^bc^	0.38 ± 0.06^bc^	0.32 ± 0.05^bc^	0.00 ± 0.00^c^	0.95 ± 0.09^a^	0.76 ± 0.11^ab^	0.51 ± 0.24^abc^	0.59 ± 0.47^ab^	0.58 ± 0.23^abc^	0.47 ± 0.08^abc^
Isopulegyl acetate*	1284	1.67 ± 0.31^b^	1.83 ± 0.08^ab^	1.33 ± 0.30^bc^	2.37 ± 0.10^a^	1.36 ± 0.06^bc^	1.44 ± 0.03^bc^	1.74 ± 0.21^b^	0.59 ± 0.16^d^	0.50 ± 0.05^d^	1.00 ± 0.01^ cd^	0.31 ± 0.41^d^	0.53 ± 0.16^d^
Bornyl acetate	1290	3.60 ± 0.14^bcd^	4.52 ± 0.75^ab^	2.98 ± 0.13^cde^	5.75 ± 0.56^a^	4.62 ± 0.49^ab^	4.11 ± 0.49^bc^	4.10 ± 0.53^bc^	2.65 ± 0.59^de^f	1.95 ± 0.33^e^f	2.12 ± 0.12^de^f	1.60 ± 0.33^e^f	1.23 ± 0.37f.
Lavandulyl acetate	1295	2.61 ± 0.46^ab^	1.00 ± 0.32^de^f	0.26 ± 0.23 fg	3.33 ± 0.18^a^	1.89 ± 0.34^bc^	1.44 ± 0.31^ cd^	2.09 ± 0.27^bc^	0.65 ± 0.14^d^-^g^	0.28 ± 0.12 fg	1.41 ± 0.14^cde^	0.42 ± 0.56^e^f^g^	0.00 ± 0.00^ g^
Sesquiterpene hydrocarbons													
Cycloisosativene	1370	0.68 ± 0.15^bc^	0.79 ± 0.18^b^	0.41 ± 0.12^bcd^	1.89 ± 0.18^a^	0.67 ± 0.16^bc^	0.26 ± 0.46^bcd^	0.65 ± 0.04^bc^	0.14 ± 0.13^ cd^	0.00 ± 0.00^d^	0.34 ± 0.11^bcd^	0.00 ± 0.00^d^	0.00 ± 0.00^d^
Sativene	1393	0.44 ± 0.12^a^	0.31 ± 0.23^ab^	0.00 ± 0.00^c^	0.17 ± 0.07^bc^	0.00 ± 0.00^c^	0.00 ± 0.00^c^	0.29 ± 0.05^ab^	0.00 ± 0.00^c^	0.00 ± 0.00^c^	0.04 ± 0.03^bc^	0.00 ± 0.00^c^	0.00 ± 0.00^c^
γ-Muurolene	1481	0.13 ± 0.04^a^	0.00 ± 0.00^c^	0.00 ± 0.00^c^	0.09 ± 0.07^ab^	0.00 ± 0.00^c^	0.00 ± 0.00^c^	0.13 ± 0.02^a^	0.00 ± 0.00^c^	0.00 ± 0.00^c^	0.01 ± 0.01^bc^	0.00 ± 0.00^c^	0.00 ± 0.00^c^
Germacrene D	1485	0.00 ± 0.00^a^	0.00 ± 0.00^a^	0.00 ± 0.00^a^	0.13 ± 0.14^a^	0.00 ± 0.00^a^	0.00 ± 0.00^a^	0.04 ± 0.03^a^	0.00 ± 0.00^a^	0.00 ± 0.00^a^	0.01 ± 0.01^a^	0.00 ± 0.00^a^	0.00 ± 0.00^a^
α-Selinene	1498	0.31 ± 0.04^a^-^d^	0.15 ± 0.25^bcd^	0.28 ± 0.25^a-d^	0.54 ± 0.10^a^	0.00 ± 0.00^d^	0.00 ± 0.00^d^	0.42 ± 0.09^abc^	0.17 ± 0.16^a^-^d^	0.00 ± 0.00^d^	0.33 ± 0.06^a^-^d^	0.52 ± 0.10^ab^	0.00 ± 0.00^ cd^
α-Muurolene	1503	0.16 ± 0.03^ab^	0.26 ± 0.16^a^	0.08 ± 0.13^ab^	0.16 ± 0.14^ab^	0.00 ± 0.00^b^	0.00 ± 0.00^b^	0.15 ± 0.01^ab^	0.00 ± 0.00^b^	0.00 ± 0.00^b^	0.04 ± 0.03^ab^	0.00 ± 0.00^ab^	0.00 ± 0.00^ab^
γ-Cadinene	1518	0.33 ± 0.08^bc^	0.30 ± 0.05^bcd^	0.19 ± 0.08^cde^	0.75 ± 0.06^a^	0.11 ± 0.12^cde^	0.00 ± 0.00^e^	0.46 ± 0.14^b^	0.19 ± 0.17^cde^	0.00 ± 0.00^e^	0.14 ± 0.02^cde^	0.03 ± 0.01^de^	0.00 ± 0.00^e^
* cis*-Calamenene	1528	1.42 ± 0.57^abc^	0.87 ± 0.34^bcd^	0.68 ± 0.14^bcd^	2.34 ± 0.66^a^	0.71 ± 0.12^bcd^	0.83 ± 0.07^bcd^	0.98 ± 0.15^bcd^	0.36 ± 0.09^d^	0.36 ± 0.01^d^	1.71 ± 0.20^ab^	0.66 ± 0.11^bcd^	0.41 ± 0.37^ cd^
α-Calacorene	1548	0.76 ± 0.11^abc^	0.74 ± 0.18^abc^	0.32 ± 0.22^b^-^e^	0.54 ± 0.02^a-d^	0.15 ± 0.25^de^	0.00 ± 0.00^e^	0.56 ± 0.15^a^-^d^	0.28 ± 0.24^cde^	0.48 ± 0.06^b-e^	1.09 ± 0.08^a^	0.88 ± 0.16^ab^	0.76 ± 0.37^abc^
Cadalene	1679	0.81 ± 0.09^c^	1.10 ± 0.37^bc^	0.92 ± 0.11^bc^	1.56 ± 0.20^bc^	1.48 ± 0.38^bc^	0.77 ± 0.48^c^	0.99 ± 0.19^bc^	1.23 ± 0.15^bc^	0.67 ± 0.32^c^	1.93 ± 0.31^b^	3.41 ± 0.57^a^	1.09 ± 0.42^bc^
β-Eudesmene	1490	1.46 ± 0.24^a^	1.17 ± 0.89^ab^	0.00 ± 0.00^d^	1.85 ± 0.28^a^	0.48 ± 0.11^bcd^	0.00 ± 0.00^d^	1.23 ± 0.07^ab^	0.18 ± 0.16^ cd^	0.00 ± 0.00^d^	1.11 ± 0.13^abc^	0.89 ± 0.04^a^-^d^	0.00 ± 0.00^ cd^
Oxygenated sesquiterpenes													
Palustrol	1572	0.22 ± 0.03^ab^	0.34 ± 0.07^a^	0.00 ± 0.00^b^	0.21 ± 0.12^ab^	0.11 ± 0.19^ab^	0.00 ± 0.00^b^	0.24 ± 0.04^ab^	0.09 ± 0.16^ab^	0.00 ± 0.00^b^	0.36 ± 0.10^a^	0.38 ± 0.06^a^	0.00 ± 0.00^b^
Caryophyllene oxide	1586	0.47 ± 0.05^a-d^	0.77 ± 0.31^a^	0.42 ± 0.10^a-d^	0.59 ± 0.04^a^-^d^	0.55 ± 0.03^a^-^d^	0.25 ± 0.44^a^-^d^	0.63 ± 0.11^abc^	0.63 ± 0.10^abc^	0.70 ± 0.04^ab^	0.13 ± 0.01^bcd^	0.06 ± 0.05^ cd^	0.00 ± 0.00^d^
Viridiflorol	1595	1.94 ± 0.14^c^	2.73 ± 0.70^bc^	2.26 ± 0.58^c^	2.43 ± 0.23^bc^	2.66 ± 0.29^bc^	3.65 ± 0.08^abc^	2.17 ± 0.17^c^	2.06 ± 1.69^c^	3.56 ± 0.42^abc^	3.04 ± 0.65^abc^	4.53 ± 0.63^ab^	5.19 ± 0.42^a^
Torreyol	1647	0.28 ± 0.03^b^	0.32 ± 0.01^ab^	0.10 ± 0.08^c^	0.32 ± 0.01^ab^	0.35 ± 0.03^ab^	0.00 ± 0.00^c^	0.27 ± 0.04^b^	0.33 ± 0.05^ab^	0.00 ± 0.00^c^	0.32 ± 0.06^ab^	0.43 ± 0.11^a^	0.00 ± 0.00^c^
α-Cadinol	1660	3.48 ± 0.41^a^	3.66 ± 0.40^a^	2.75 ± 1.30^a^	3.37 ± 0.85^a^	3.83 ± 1.00^a^	5.31 ± 1.81^a^	3.45 ± 1.17^a^	4.14 ± 0.85^a^	4.72 ± 1.58^a^	1.96 ± 0.26^a^	3.17 ± 0.23^a^	3.52 ± 0.76^a^
Hexahydrofarnesyl acetone	1850	0.05 ± 0.06^a^	0.48 ± 0.20^a^	0.37 ± 0.22^a^	0.20 ± 0.11^a^	0.00 ± 0.00^a^	0.74 ± 0.64^a^	0.17 ± 0.03^a^	0.32 ± 0.35^a^	0.69 ± 0.61^a^	0.11 ± 0.13^a^	0.04 ± 0.06^a^	0.00 ± 0.00^a^
Non-terpenes													
2-Methyltetrahydrofuran-3-one	< 900	0.00 ± 0.00^b^	0.00 ± 0.00^b^	0.29 ± 0.08^a^	0.00 ± 0.00^b^	0.00 ± 0.00^b^	0.00 ± 0.00^b^	0.00 ± 0.00^b^	0.00 ± 0.00^b^	0.00 ± 0.00^b^	0.00 ± 0.00^b^	0.00 ± 0.00^b^	0.00 ± 0.00^b^
2(5H)-Furanone, 5,5-dimethyl-	961	0.41 ± 0.03^a^	0.44 ± 0.09^a^	0.45 ± 0.14^a^	0.00 ± 0.00^b^	0.00 ± 0.00^b^	0.00 ± 0.00^b^	0.00 ± 0.00^b^	0.00 ± 0.00^b^	0.00 ± 0.00^b^	0.06 ± 0.08^b^	0.00 ± 0.00^b^	0.00 ± 0.00^b^
4-Methyl-2(5H)-furanone*	1055	0.00 ± 0.00^b^	0.00 ± 0.00^b^	0.12 ± 0.11^a^	0.00 ± 0.00^b^	0.00 ± 0.00^b^	0.00 ± 0.00^b^	0.00 ± 0.00^b^	0.00 ± 0.00^b^	0.00 ± 0.00^b^	0.00 ± 0.00^b^	0.00 ± 0.00^b^	0.00 ± 0.00^b^
3-Acetyl-2,4-dimethylfuran	1096	0.83 ± 0.13^bc^	0.82 ± 0.14^bc^	0.79 ± 0.08^bc^	0.42 ± 0.07^d^	0.69 ± 0.16^ cd^	0.90 ± 0.16^bc^	0.57 ± 0.03^ cd^	0.72 ± 0.08^ cd^	0.91 ± 0.17^bc^	1.16 ± 0.08^b^	1.64 ± 0.06^a^	1.86 ± 0.10^a^
Benzyl alcohol	1043	0.00 ± 0.00^b^	0.24 ± 0.06^a^	0.19 ± 0.10^a^	0.00 ± 0.00^b^	0.00 ± 0.00^b^	0.00 ± 0.00^b^	0.00 ± 0.00^b^	0.00 ± 0.00^b^	0.00 ± 0.00^b^	0.00 ± 0.00^b^	0.00 ± 0.00^b^	0.00 ± 0.00^b^
4-Methoxy-benzaldehyde	1265	0.00 ± 0.00^b^	0.00 ± 0.00^b^	0.36 ± 0.12^a^	0.00 ± 0.00^b^	0.00 ± 0.00^b^	0.00 ± 0.00^b^	0.00 ± 0.00^b^	0.00 ± 0.00^b^	0.00 ± 0.00^b^	0.00 ± 0.00^b^	0.00 ± 0.00^b^	0.00 ± 0.00^b^
2-Phenylethanol	1125	0.00 ± 0.00^b^	0.00 ± 0.00^b^	0.73 ± 0.68^a^	0.00 ± 0.00^b^	0.00 ± 0.00^b^	0.00 ± 0.00^b^	0.00 ± 0.00^b^	0.00 ± 0.00^b^	0.00 ± 0.00^b^	0.00 ± 0.00^b^	0.00 ± 0.00^b^	0.00 ± 0.00^b^
Ethanol	< 900	0.00 ± 0.00^c^	0.00 ± 0.00^c^	0.63 ± 0.22^b^	0.05 ± 0.09^c^	0.00 ± 0.00^c^	0.00 ± 0.00^c^	0.00 ± 0.00^c^	0.55 ± 0.09^b^	0.75 ± 0.22^b^	0.17 ± 0.03^c^	0.83 ± 0.11^b^	1.79 ± 0.04^a^
Acetic acid	< 900	1.27 ± 0.38^bc^	6.12 ± 4.32^b^	15.04 ± 4.23^a^	0.00 ± 0.00^c^	0.00 ± 0.00^c^	0.76 ± 1.32^bc^	0.00 ± 0.00^c^	0.14 ± 0.25^c^	1.99 ± 1.46^bc^	0.04 ± 0.01^c^	1.50 ± 0.19^bc^	5.43 ± 0.54^bc^
Propanoic acid	< 900	0.00 ± 0.00^b^	0.30 ± 0.30^a^	0.28 ± 0.06^a^	0.00 ± 0.00^b^	0.00 ± 0.00^b^	0.00 ± 0.00^b^	0.00 ± 0.00^b^	0.00 ± 0.00^b^	0.00 ± 0.00^b^	0.00 ± 0.00^b^	0.00 ± 0.00^b^	0.00 ± 0.00^b^
2-Methypropanoic acid	< 900	0.05 ± 0.02^a^	0.25 ± 0.31^a^	0.21 ± 0.03^a^	0.00 ± 0.00^a^	0.00 ± 0.00^a^	0.00 ± 0.00^a^	0.00 ± 0.00^a^	0.00 ± 0.00^a^	0.00 ± 0.00^a^	0.00 ± 0.00^a^	0.00 ± 0.00^a^	0.00 ± 0.00^a^
Butanoic acid	< 900	0.00 ± 0.00^a^	0.07 ± 0.08^a^	0.06 ± 0.10^a^	0.00 ± 0.00^a^	0.00 ± 0.00^a^	0.00 ± 0.00^a^	0.00 ± 0.00^a^	0.00 ± 0.00^a^	0.00 ± 0.00^a^	0.00 ± 0.00^a^	0.00 ± 0.00^a^	0.00 ± 0.00^a^
2-Methylbutanoic acid	< 900	0.01 ± 0.01^b^	0.10 ± 0.04^a^	0.10 ± 0.03^a^	0.00 ± 0.00^b^	0.00 ± 0.00^b^	0.00 ± 0.00^b^	0.00 ± 0.00^b^	0.00 ± 0.00^b^	0.00 ± 0.00^b^	0.00 ± 0.00^b^	0.00 ± 0.00^b^	0.00 ± 0.00^b^
3-Methylbut-2-enoic acid	< 900	0.00 ± 0.00^b^	0.12 ± 0.07^a^	0.11 ± 0.06^a^	0.00 ± 0.00^b^	0.00 ± 0.00^b^	0.00 ± 0.00^b^	0.00 ± 0.00^b^	0.00 ± 0.00^b^	0.00 ± 0.00^b^	0.00 ± 0.00^b^	0.00 ± 0.00^b^	0.00 ± 0.00^b^
γ-Butyrolactone	925	0.00 ± 0.00^b^	0.00 ± 0.00^b^	0.25 ± 0.05^a^	0.00 ± 0.00^b^	0.00 ± 0.00^b^	0.00 ± 0.00^b^	0.00 ± 0.00^b^	0.00 ± 0.00^b^	0.00 ± 0.00^b^	0.00 ± 0.00^b^	0.00 ± 0.00^b^	0.00 ± 0.00^b^
Hexan-2,5-dione	938	0.00 ± 0.00^b^	0.00 ± 0.00^b^	0.12 ± 0.04^a^	0.00 ± 0.00^b^	0.00 ± 0.00^b^	0.00 ± 0.00^b^	0.00 ± 0.00^b^	0.00 ± 0.00^b^	0.00 ± 0.00^b^	0.00 ± 0.00^b^	0.00 ± 0.00^b^	0.00 ± 0.00^b^
Hexanoic acid	981	0.00 ± 0.00^b^	0.17 ± 0.06^ab^	0.33 ± 0.27^a^	0.00 ± 0.00^b^	0.00 ± 0.00^b^	0.00 ± 0.00^b^	0.00 ± 0.00^b^	0.00 ± 0.00^b^	0.00 ± 0.00^b^	0.00 ± 0.00^b^	0.00 ± 0.00^b^	0.00 ± 0.00^b^
Oct-1-en-3-ol	985	0.13 ± 0.01^a^	0.00 ± 0.00^b^	0.00 ± 0.00^b^	0.00 ± 0.00^b^	0.00 ± 0.00^b^	0.00 ± 0.00^b^	0.00 ± 0.00^b^	0.00 ± 0.00^b^	0.00 ± 0.00^b^	0.00 ± 0.00^b^	0.00 ± 0.00^b^	0.00 ± 0.00^b^
3-Methylbenzaldehyde	1090	0.00 ± 0.00^a^	0.00 ± 0.00^a^	0.12 ± 0.21^a^	0.00 ± 0.00^a^	0.00 ± 0.00^a^	0.00 ± 0.00^a^	0.00 ± 0.00^a^	0.00 ± 0.00^a^	0.00 ± 0.00^a^	0.00 ± 0.00^a^	0.00 ± 0.00^a^	0.00 ± 0.00^a^
6-Methyl-3,5-heptadien-2-one	1109	0.18 ± 0.06^ab^	0.34 ± 0.05^a^	0.29 ± 0.15^a^	0.00 ± 0.00^b^	0.00 ± 0.00^b^	0.00 ± 0.00^b^	0.08 ± 0.01^b^	0.07 ± 0.12^b^	0.00 ± 0.00^b^	0.17 ± 0.06^ab^	0.01 ± 0.02^b^	0.00 ± 0.00^b^
O ct-1-en-3-yl acetate	1117	0.11 ± 0.03^abc^	0.14 ± 0.04^ab^	0.04 ± 0.07^bcd^	0.21 ± 0.02^a^	0.00 ± 0.00^d^	0.00 ± 0.00^d^	0.17 ± 0.03^a^	0.04 ± 0.06^ cd^	0.00 ± 0.00^d^	0.01 ± 0.01^ cd^	0.00 ± 0.00^ cd^	0.00 ± 0.00^ cd^
Methylacetophe-none	1189	0.62 ± 0.13^ab^	0.73 ± 0.05^a^	0.41 ± 0.23^bc^	0.00 ± 0.00^d^	0.00 ± 0.00^d^	0.00 ± 0.00^d^	0.28 ± 0.04^ cd^	0.29 ± 0.04^ cd^	0.00 ± 0.00^d^	0.28 ± 0.02^ cd^	0.29 ± 0.30^bcd^	0.00 ± 0.00^d^
Heptadecane	1700	0.13 ± 0.03^a^	0.12 ± 0.02^a^	0.00 ± 0.00^b^	0.05 ± 0.05^b^	0.00 ± 0.00^b^	0.00 ± 0.00^b^	0.05 ± 0.03^b^	0.00 ± 0.00^b^	0.00 ± 0.00^b^	0.01 ± 0.01^b^	0.00 ± 0.00^b^	0.00 ± 0.00^b^
α-Isophorone	1086	0.75 ± 0.23^b^	0.69 ± 0.31^b^	0.54 ± 0.23^b^	0.45 ± 0.08^b^	0.56 ± 0.09^b^	0.78 ± 0.07^b^	0.56 ± 0.09^b^	0.64 ± 0.07^b^	0.62 ± 0.15^b^	1.03 ± 0.15^ab^	1.10 ± 0.08^ab^	1.65 ± 0.58^a^

Means ± standard deviation followed by different letters in the same row differ significantly, based on Tukey’s test at *p* < 0.05.

The initial headspace analysis (start/0 month) of control extract and DESs-CO_2_ samples indicated that there was a predominant presence of oxygenated terpenes: monoterpenes (67.33–77.50% DVB/CAR/PDMS and 67.45–74.63% PDMS/DVB), and sesquiterpenes (5.64–8.57% DVB/CAR/PDMS and 5.91–7.10% PDMS/DVB). Terpene hydrocarbons were less present in the samples: sesquiterpene hydrocarbons (6.47–12.33% DVB/CAR/PDMS and 5.92–10.02% PDMS/DVB) and monoterpene hydrocarbons (0–1.94% DVB/CAR/PDMS and 0–1.29% PDMS/DVB). The difference in percentual abundance of the components identified using different fibers was caused by the fibers’ different polarities. PDMS/DVB is characterized by lower absorption of low molecular terpene components and polar alcohols^27^. However, their distribution and the trend of components identified using two fibers during storage was similar. In addition, the differences between the profiles of the control and DESs-CO_2_ samples at the start (0 month) were the result of different batches. The used herbal material was a commercial sample, therefore, the differences in material caused by season pickings and localities were possible, hence all samples were monitored from 0 to 6 months. Furthermore, significant fluctuations in the chemical profile of *L. stoechas* were reported in literature due to different environmental conditions and geolocation, and picking conditions. However, the predominant presence of oxygenated monoterpenes and low presence of hydrocarbons were in alignment with the literature^18,28,29^.

Over time, changes were observed in the chemical profile of the samples. Namely, there was a decrease in the presence or even disappearance of certain components, as well as formation of new compounds. According to Mehdizadeh et al.^20^, the reasons behind these changes could be evaporation, oxidation, and other chemical transformations which can lead to significant changes in sensory characteristics and biological activities.

The abundance of components that belong to terpene hydrocarbons decreased over time, and monoterpene hydrocarbons nearly disappeared, while there was a significant drop in sesquiterpene hydrocarbons during 6 months in all samples (control extract from 6.47 to 4.15% (DVB/CAR/PDMS) and from 6.48 to 2.87% (PDMS/DVB); sample Bet:EG from 12.33 to 4.35% (DVB/CAR/PDMS) and from 10.02 to 1.87% (PDMS/DVB); Bet:Gly from 7.41 to 1.90% (DVB/CAR/PDMS) and from 5.92 to 1.52% (PDMS/DVB); Gly:Glu from 7.77 to 3.79% (DVB/CAR/PDMS) and from 6.77 to 2.25% (PDMS/DVB))*.* The trend of the decrease in the presence of hydrocarbons was previously observed during storage of essential oil of different aromatic herbs^30,31^. Rowshan et al.^32^ reported evaporation as the main cause of the decrease in the presence of hydrocarbons during storage due to their low boiling point. Additionally, hydrocarbons, especially monoterpene hydrocarbons, are characterized by instability because of their sensitivity to heat and light, hence they can degrade and cause unpleasant smells. A common practice during industrial production of essential oil is to remove hydrocarbons (deterpenation) to preserve the quality of the product^33^.

Oxygenated terpenes are characterized by higher stability compared to hydrocarbons, and the decrease in presence of lower molecular weight hydrocarbons could be the cause of the increased percentage of oxygenated monoterpenes in Bet:EG and Bet:Gly in the first 3 months. After 6 months, there was a decrease in oxygenated monoterpenes in Bet:EG and Bet:Gly, which could be due to the increased presence of oxygenated sesquiterpenes. Namely, due to their higher molecular weight and boiling point, oxygenated sesquiterpenes are more stable than oxygenated monoterpenes, therefore, the increase in their amount during storage was determined. In the control and sample Gly:Glu, the decrease in the amount of oxygenated monoterpenes during storage was recorded. This amount was impacted by the increase in the amount of non-terpene compounds. In the non-terpene group, some of the present components were characteristic of the volatile profiles of lavender which participate in the formation of aroma, such as oct-1-en-3-yl acetate^34^, as well as the components which were formed during storage.

The dominant aromatic volatile components in the samples were fenchone (16.10–35.27% DVB/CAR/PDMS and 15.38–37.56% PDMS/DVB) and verbenone (4.10–20.74% DVB/CAR/PDMS and 3.58–24.38% PDMS/DVB) from the group of oxygen-containing monoterpenes. From the group of oxygenated sesquiterpenes, viridiflorol and α-cadinol were the most abundant with 1.61–6.09% (DVB/CAR/PDMS)/1.94–5.19% (PDMS/DVB) and 2.29–6.64% DVB/CAR/PDMS/1.96–5.31% (PDMS/DVB), respectively. Among monoterpene hydrocarbons, α-pinene, camphene *p*-cymene, and limonene were detected (below 1%). Among sesquiterpene hydrocarbons, the dominant ones were *cis*-calamenene, cadelene, and *β*-eudesmene.

As volatile components are sensitive and have a tendency towards different biotransformations, it is difficult to precisely establish the processes of their conversion over time, because the transformations can be easily induced. Extracts represent mixtures of a high number of components, hence the transformation of a single component can initiate the transformation of others. Moreover, the degradation can take place in various degradation pathways, such as: oxidative degradation, C–C bond cleavage, elimination, hydrolysis, or thermal rearrangement^35^. According to Turek and Stintzing^19^, significant oxidative processes occur during storage. In addition, the authors monitored changes in lavender oil during 72 weeks in storage with the presence of atmospheric oxygen at 23 °C in the dark and at 23 °C and 38 °C under cool white light. The study further suggested that due to elevated temperature, the formation of hydroperoxides occurred through radical reaction with oxygen during the first stage of autoxidation, which were further decomposed into stable oxidation products such as ketones, alcohols, epoxides, or acids^19^.

Hydrocarbons are susceptible to evaporation which could be the cause of the decrease in their amount over time. Also, because of their propensity for oxidation, hydrocarbons can form radicals stabilized by conjugated double bonds or by isomerization to tertiary radicals. Furthermore, oxygenated terpenes can directly convert into ketones, acids, and aldehydes because of oxidation^19^. Due to their chemical similarity, volatile compounds within the same group can transform easily into each other, which can be initiated by different external or internal factors^19^. The increase in the percentage of fenchone over time could be due to the oxidation of fenchol. Also, fenchone is an isomer of borneol and these two monoterpenes can transform into each other by oxidoreductive reactions. In addition, bornyl acetate can be created by the conversion of camphor and borneol^36^. The transformation of *α*-pinene can form verbenone and α-campholene aldehyde, while verbenone can be formed as a degradational product of camphene^35^. Dominant oxygenated sesquiterpenes such as viridiflorol and α-cadinol increased in amount over time due to their higher stability.

In DES samples, a decrease in relative percentage of bornyl acetate and lavandulyl acetate could be noted in both fibers, most likely due to the hydrolysis of these esters. In the control group, there was drop in the presence of tertiary alcohol linalool until its disappearance after 6 months, probably caused by oxidation indicated by the increasing abundance of its derivatives *trans*-linalool oxide and lavender lactone. The identical trend was recorded in the sample Gly:Glu, while in Bet:Gly the stability of linalool was better. In Bet:EG, such behaviour was not recorded, hence the presence of linalool increased over time. Moreover, the lavender lactone was not present in the sample, while *trans*-linalool oxide was present in a lower percentage compared to remaining samples and control, which indicates higher stability of linalool in the Bet:EG mixture.

Furthermore, the boiling point of organic compounds can affect their stability, that is, decelerate the degradation of components^37^. For instance, among the dominant sesquiterpene hydrocarbons, *cis*-calamenene, cadelene, and *β*-eudesmene, the fastest degradation was that of *β*-eudesmene, which also presents the lowest boiling point, was not detected in the samples after 6 months. A slower degradation was recorded with *cis*-calamenene, which has a higher boiling point than *β*-eudesmene. The highest stability was established with cadalene, which demonstrated growth in all samples after 3 months and possesses the highest boiling point.

Significant changes in the relative presence of components were determined for non-terpene components which included 26 components belonging to different classes. In the control sample, 15 new compounds were detected after storage and were not present in the DESs-CO_2_ samples. These newly formed compounds significantly impacted the relative percentual distribution of volatile components and could potentially be the indicator of the control’s decreased stability.

The relative share of organic acids in the control significantly increased during storage, and after 3 months it was 4.14% (DVB/CAR/PDMS) and 7.14% (PDMS/DVB), while after 6 months it increased to 10.86% (DVB/CAR/PDMS) and 16.14% (PDMS/DVB). The dominant organic acid was acetic acid with a multifold increase in its abundance in the control during 6 months (from 1.27 to 15.04% PDMS/DVB fiber and from 1.17 to 10.05% DVB/CAR/PDMS), which matched the mild off-smell developed in the control extract. Apart from acetic acid, other organic acids were detected in the control extract such as butanoic, 3-methylbutanoic, propanoic, and 2-methylpropanoic acid which could participate in the formation of the characteristic unpleasant, rancid, sweaty, and cheese-like odour and they can represent products of fermentation^38,39^.

In DESs-CO_2_ samples, only acetic acid from the group organic volatile acids was detected in a significantly lower percentage compared to the control. In the sample Gly:Glu, the increase in the percentage of acetic acid to 3.86% (DVB/CAR/PDMS) and 5.43% (PDMS/DVB) was determined. After 6 months, the Bet:Gly sample contained 0.87% (DVB/CAR/PDMS) and 1.99% (PDMS/DVB) of acetic acid. In the Bet:EG sample, with the use of DVB/CAR/PDMS fiber, the presence of acetic acid was not detected after 6 months, and with the PDMS/DVB fiber 0.76% of acetic acids was determined.

In addition, in the control sample, the presence of ethanol increased over time. Ethanol was also detected in the samples Bet:Gly and Gly:Glu, while in Bet:EG, its presence was not established. This alcohol is considered as one of the primary by-products of fermentation and with its conversion, there could be an increase in the amount of acetic acid in the control. This conversion was reported to occur during the beer fermentation process^40^. Moreover, other compounds that can be a part of the fermentation such as organic acids, alcohols, aldehydes, and ketones, were detected in the control samples. Volatile acids could be potentially associated with fermentation, and oxidation as well, because secondary oxidation of aldehydes can also form short chain acids^39^. Therefore, the changes in relative percentage of these components accompanied with the alteration in smell indicated the decreased stability of the control samples.

By using the PDMS/DVB fiber in the control after 6 months, the presence of furanones: 4-methyl-2(5H)-furanone and 2-methyltetrahydrofuran-3-one was detected, and both were not present in DESs-CO_2_. 2-Methyltetrahydrofuran-3-one is a characteristic constituent of the aroma of roasted coffee, while according to Pinto et al.^41^, it represents an oxidation marker of wine and its presence in wine is connected with undesirable notes which are developed during oxidation. Also, in the control, dihydrofuran—5,5-dimethyl-2(5H)-furanone was detected. 3-Acetyl-2,4-dimethylfuran was the only furan derivative identified in the control and DESs-CO_2_ samples and its relative share grew over time. The total percentage of furan derivatives in Bet:EG and Bet:Gly was below 1%, while in the control and Gly:Glu it was 0.99% (DVB/CAR/PDMS) and 1.70% (PDMS/DVB) and 2.06% (DVB/CAR/PDMS) and 1.86% (PDMS/DVB), respectively.

In the control sample, benzyl alcohol was detected after 3 months and its abundance increased with time (not in DESs-CO_2_). The increase in benzyl alcohol was connected with off-odours which developed as the result of 4 weeks of storage of *Durio zibethinus* cv. D24 fruit at 4 °C^42^. Additionally, aldehyde compounds such as benzaldehyde, 3-methylbenzaldehyde, and 4-methoxybenzaldehyde were identified only in the control extract after 3 and 6 months, while at the beginning, they were not discovered in the headspace profile. A very low presence of benzaldehyde (DVB/CAR/PDMS) was determined in the control, which can occur as the result of lipid oxidation^43^, while 4-methoxybenzaldehyde could be the oxidation product connected with benzyl alcohol or the decomposition of lignin.

DESs can impact the solubility and stability of compounds by establishing or preventing interactions between molecules^12^ by: (i) the internal structure of DES that forms a polymer-like matrix, and the solute can be dissolved into the space/hole of this network (the hole or liquid crystal theory); as the component ratio changes, the inner characteristics such as the size and shape of the matrix may be altered significantly, and (ii) the binding theory, according to which intermolecular interactions occur between DES components and target solutes, making the components part of the DES matrix. In addition, this can lead to the change in properties of target compounds such as solubility and miscibility.

Based on the obtained results, it can be concluded that investigated samples DESs-CO_2_ were more stable than the CO_2_ control. Among them, Bet:EG stood out as the most adequate for maintaining the stability of the HS components of *L. stoechas*. One of the explanations could be that DESs acted as stabilizing media, served as protection against light and oxygen, reducing the oxidative degradation of the extracts’ components. This explanation is in agreement with the conclusions made by Bitterling et al.^44^ who established that essential oil could have the role of a barrier and protector from light and oxygen. Essential oil was significantly more adequate for preserving the quality of terpene compared to pure terpene compounds. The presence of numerous components in the essential oil which possess antioxidative and stabilizing properties could contribute to the deceleration of the oxidative processes. The components prone to oxidation, by the transfer of reactive oxygen, could initiate the oxidation of other components which are less sensitive. However, the presence of other components can prevent the changes and impact the preservation of stability by postponing or interrupting the chain reactions of oxidation^19^. This stabilising effect can be additionally intensified in DESs. It was reported that betaine could protect metabolites such as proteins against denaturation and aggregation^16^.

The molecular structure and the composition of the matrix impact the stability of components^45^. It is evident that the composition of DES had a significant impact. Terpene structure has lipophilic characteristics, while the polar functional group is accountable for hydrophilic properties^46^. The extract represents a mixture of different components among which were terpene hydrocarbons and oxygenated terpenes. Therefore, the extract possesses numerous constituents which belong to different classes of compounds. DES components can form additional hydrogen bonds with different organic compounds and increase their solubility^16^. Due to the presence of different classes of components with different functional groups, it can be inferred that in the mixture DES-extract, various intermolecular interactions are possible between DESs constituents and functional groups of lipophilic compounds of *L. stoechas* extract, such as carboxyl and hydroxyl groups. In this work, the DES comprised of sugar and sugar alcohol (Gly:Glu) had the least stabilising effect, while the higher stability was provided by glycerol in combination with betaine. Moreover, betaine in combination with ethylene glycol proved to be the most adequate for maintaining stability. Betaine, because of its carboxylate group is a strong hydrogen bond acceptor and can form DES with organic compounds which possess a hydrogen bond donning capability^21^. Moreover, the most adequate DES, Bet:EG was the least polar (50.75 kcal mol^−1^), while Bet:Gly and Gly:Glu were more polar with values 49.98 i 49.04 kcal mol^−1^, respectively.

Furthermore, the viscosity of the systems may also play an important role in the stabilization of the molecules. According to Dai et al.^13^, higher viscosity reduces the molecular mobility and, in that way, enables a more stable bond between analytes and DES components. In our study, such impact of viscosity on the stability of *L. stoechas* headspace components was not observed. Namely, the least viscous Bet:EG (48.63 ± 0.42 mPa.s at 30 °C) demonstrated to be the most adequate for the preservation of stability, while the changes were more prominent in the more viscous Bet:Gly (1250.1 ± 0.00 mPa s at 30 °C) and the most prominent in the most viscous sugar-based Gly:Glu (5443.90 ± 46.53 mPa s at 30 °C). Higher stability in the less viscous DES represents a huge advantage because it enables easier manipulation and work.

### Chemometrics of the extracts’ composition

#### Hierarchical clustering

**Figure 3:**
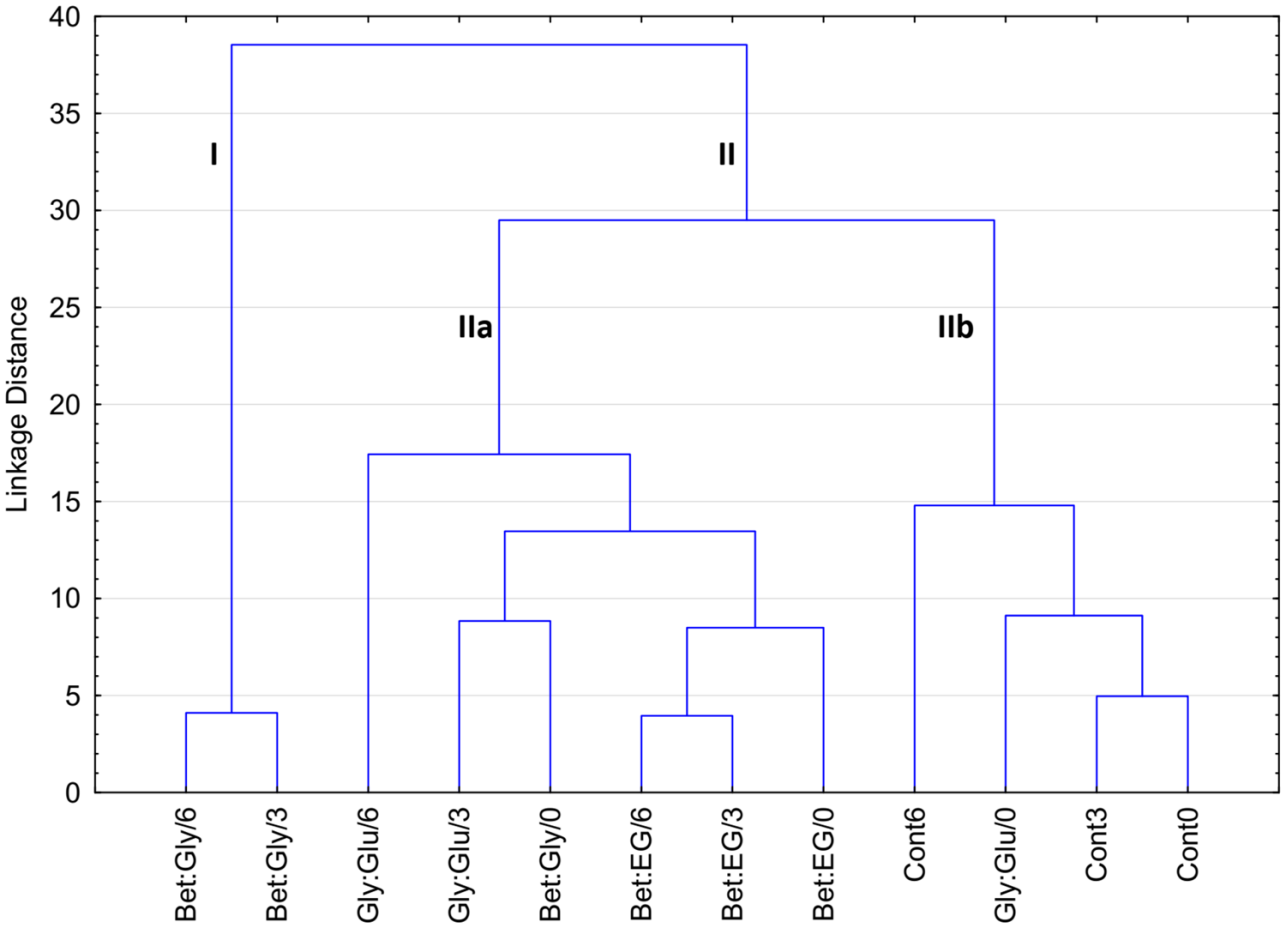
Dendrogram of the HCA of the analysed samples in terms of the chemical composition. The figure was created with TIBCO Software Inc. (2020). Data Science Workbench, version 14. http://tibco.com.

The chemometric approach was applied to visually distinguish the patterns, groupings, similarities, and differences between the samples during different storage time. The preliminary pattern recognition analysis of the samples was conducted by HCA. The obtained results are presented in Fig. 3 as a dendrogram. Two main clusters are observable. The first cluster (I) contains Bet:Gly/6 and Bet:Gly/3 samples marking them as the most different from the others. The second cluster (II) is divided into two sub-clusters: IIa and IIb. In the sub-cluster IIb the control samples are placed, while in the sub-cluster IIa, there are the samples Bet:EG/0, Bet:EG/3, and Bet:EG/6 clearly separated in a particular cluster so the highest similarity is determined between Bet:EG/3 and Bet:EG/6 samples, while the Bet:EG/0 is placed at a certain distance from them in terms of similarity in the space of the analysed variables. These positions of Bet:EG samples correspond to the result which shows there was a minimal formation of the components that could impair the quality of the extracts. Also, the lack of grouping of samples (control and DESs-CO_2_) at the start/0 month pointed to the differences between the samples. As previously mentioned, these differences between the samples at the start could be caused by the different batches and potential non-uniformity of commercial plant material samples. The HCA could not reveal which variables had the main influence on the presented grouping of the samples, therefore, the PCA was employed in the following step of the chemometric analysis.

#### Principal component analysis

**Figure 4:**
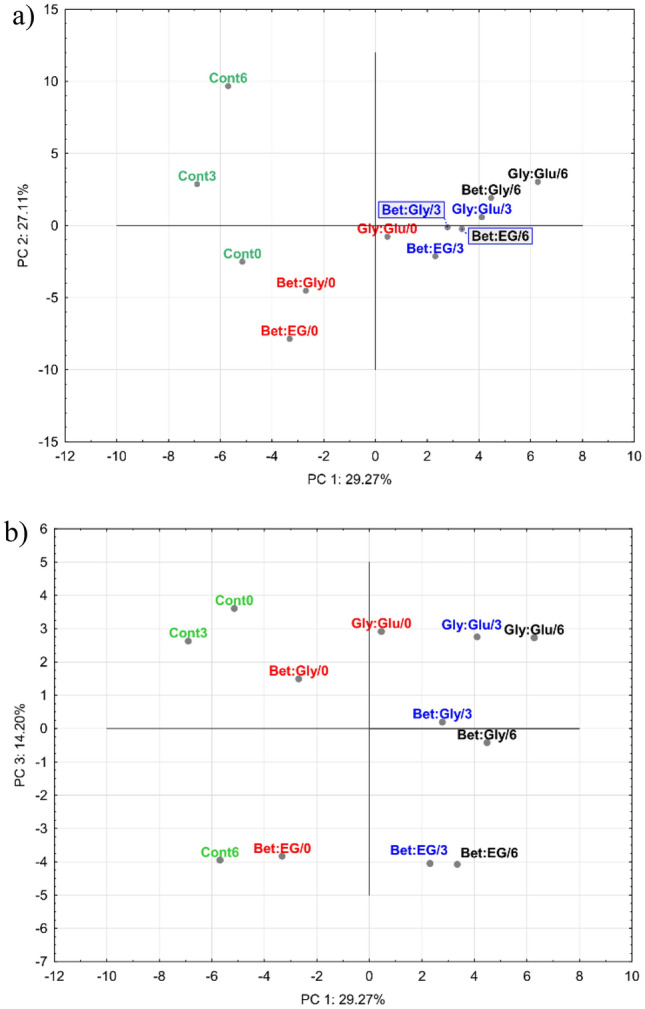
The scores plots of the samples in (**a**) PC1-PC2 space and (**b**) PC1-PC3 space. The figure was created with TIBCO Software Inc. (2020). Data Science Workbench, version 14. http://tibco.com.

PCA resulted in an 8-component model that covers up to 97.3% of total variance. All eight PCs are described by Eigenvalue greater than 1. Only the first three components were taken into account since the others were not of significance in terms of the distribution of samples on the scores plots. The PC1 takes into account 29.27% of total variance, while the PC2 considers 27.11% and PC3 14.20% of total variance. The distribution of the samples in PC1-PC2 and PC1-PC3 space is presented in Fig. 4. The corresponding loadings plots are presented in Supplementary data (Fig. S1).

On the PC1–PC2 plot, it can be seen that there is a clear separation of the control samples which are placed at the negative end of the PC1 axis. Going along the PC1 axis from its negative end towards the positive end, it can be noticed that the compounds analysed at the start of the experiment are significantly separated from the control and the samples analysed after 3 and 6 months. The separation between 3 and 6 months samples is partial since there is a certain overlap between these two groups along the PC1 axis, which corresponds with the decrease and disappearance of a significant number of hydrocarbons after 3 months. There is no particular separation of the samples along the PC2 axis. The PC1–PC3 scores plot revealed the separation of the samples in a clearer way so along the PC1 axis there is a significant separation between the control samples, which are placed at the negative end, followed by the samples analyzed at the start (0 month), 3 months, and 6 months samples. The separation of the control sample points to its significant difference compared to other samples, which aligns with the development of newly formed compounds in the extract over time. The samples analyzed after 3 and 6 months are placed much closer along the PC1 axis than the samples analyzed at the start and after 3 months. Therefore, there is a significant difference between the samples after 3 months, due to the formation of new components. The PC3 axis revealed that the samples can be also clearly separated based on the type of DES mixtures used. The samples with Bet:EG mixture are placed at the negative end of PC3 (together with Cont6). These samples are placed at high distance from the others. At the positive PC3 end there are the samples with Gly:Glu mixture together with Cont0 and Cont3 samples. The samples with Bet:Gly mixture are between the samples that are placed at the negative and the positive end of PC3 axis. Therefore, the PC1–PC3 graph reveals the significant separation of the samples based on the applied DES mixtures as well as based on the time of the storage.

#### SRD analysis

**Figure 5:**
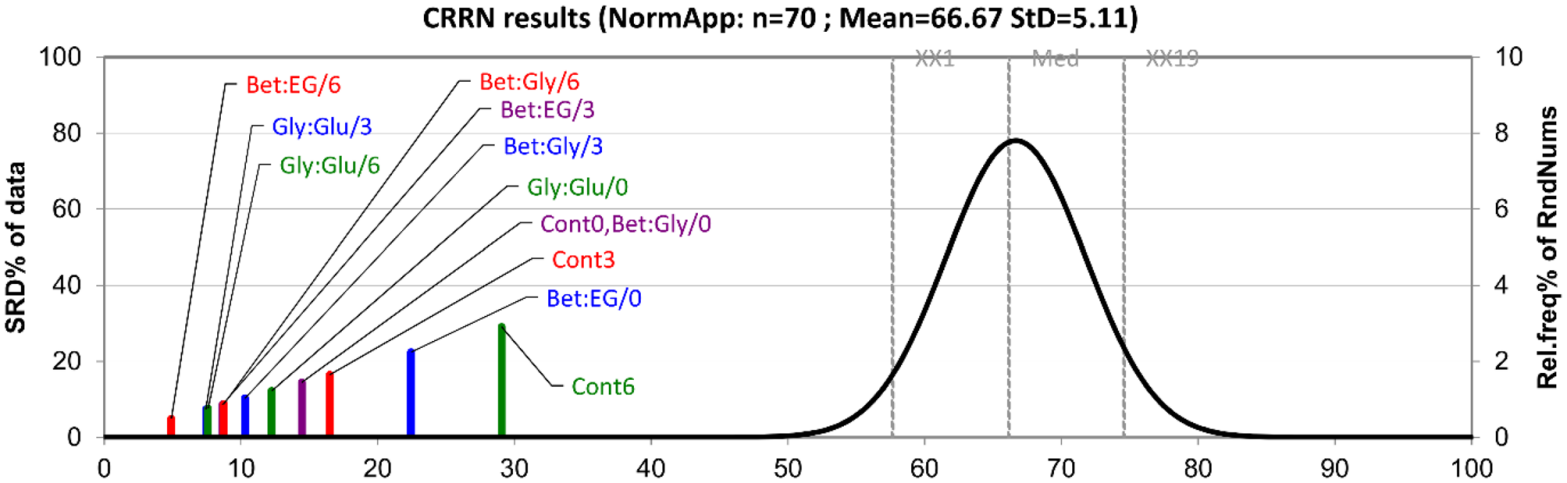
The ranking of the extracts by sum of ranking differences and comparison of ranks by random numbers with row average as a reference ranking. The statistical characteristics of Gaussian fit are the following: first icosaile (5%), XX1 = 1414; first quartile, Q1 = 1536; median, Mediana (Med) = 1622; last quartile, Q3 = 1706; last icosaile (95%), XX19 = 1828. The figure was created with Microsoft Excel, version 2305. https://www.microsoft.com/en-us/microsoft-365/excel.

The main aim of the conducted SRD analysis was to rank the samples based on the defined reference ranking (the average row values) (Fig. 5). The sample closest to the average is Bet:EG/6, followed by the samples Gly:Glu/3 and Gly:Glu/6. The samples Bet:EG/0 and Cont6 are located the farthest from the reference implying the existence of certain anomalies with these samples in terms of the chemical composition comparing to the average. Also, generally speaking, the samples analyzed after 3 and 6 months are placed closer to the reference than the DES samples analyzed at the start. The exceptions are the samples Cont3 and Cont6 where a great number of new components was formed, accounting for their further positions.

The ranking procedure was validated by sevenfold cross-validation approach and the obtained results can be considered valid. The results of the cross-validation are presented in Table 3. The normalized SRD values imply that the similar rankings of the samples were obtained in each iteration during validation.

**Table 3:** The results of sevenfold cross-validation of SRD procedure (normalized SRD values).

Sample	SRD1	SRD2	SRD3	SRD4	SRD5	SRD6	SRD7	SRD_average_
Bet:EG/6	5.3	5.3	5.1	4.3	4.8	5.0	4.3	4.9
Gly:Glu/3	9.0	9.8	6.9	7.3	6.7	6.8	5.9	7.5
Gly:Glu/6	8.9	10.2	7.1	7.4	7.6	6.0	5.6	7.5
Bet:Gly/6	10.9	11.3	8.2	8.9	7.2	6.6	7.3	8.6
Bet:EG/3	9.3	6.8	9.4	8.4	9.0	9.0	8.6	8.7
Bet:Gly/3	11.1	11.9	11.3	8.7	9.2	10.0	9.6	10.3
Gly:Glu/0	11.3	12.2	13.8	11.9	12.6	11.3	11.7	12.1
Bet:Gly/0	13.1	14.6	14.9	14.0	14.9	13.3	15.6	14.3
Cont0	14.8	12.9	16.8	14.4	14.9	14.2	13.6	14.5
Cont3	13.9	14.6	17.7	16.8	18.0	17.3	18.2	16.6
Bet:EG/0	24.8	21.1	23.0	20.0	23.6	19.1	23.7	22.2
Cont6	24.0	27.8	29.3	29.8	31.1	27.6	33.0	28.9

## Methods

### Material and chemicals

*Lavandula stoechas* L. ssp. *stoechas* flowers were purchased in Celeiro, Lisbon, Portugal. *L. stoechas* is not an endangered species, and as a commercially registered product, the collection of plant material complies with relevant institutional, national, and international guidelines and legislation. The mean particle size (0.31 ± 0.05 mm) of the material was determined using the vibration sieve sets (CISA, Cedaceria, Spain). Moisture content of the plant material (7.24%) was determined using a moisture analyser DAB (Kern, Balingen, Germany).

Betaine (≥ 99% purity), D-glucose monohydrate (≥ 97.5% purity), and Nile red were purchased from Sigma-Aldrich (St. Louis, Missouri, United States). Glycerol (99.5% purity) was purchased from Scharlau (Barcelona, Spain). Ethylene glycol (≥ 99.5% purity) and ethanol (99% purity) were obtained from Carlo Erba (Val-de-Reuil, France).

### Preparation and characterisation of DES

DES mixtures were prepared by the mixing components in the adequate molar ratio and then heated (40 °C) and stirred until a clear liquid was formed. The DESs which were applied were betaine:ethylene glycol (Bet:EG) (1:3), betaine:glycerol (Bet:Gly) (1:2), and glycerol:glucose (Gly:Glu) (4:1).

### Determination of the water content

Karl-Fischer titration using an 831 KF Coulometer with generator electrode (Metrohm, Herisau, Switzerland) was used to determine the water content of each DES. Measurements were conducted in triplicates.

### Determination of polarity by Nile red assay

The polarity of the prepared DESs was determined by using solvatochromic method with Nile red, described previously in Fernandes et al.^47^. The absorbance of the samples was obtained using a UV-spectrophotometer (GENESYS 50, Thermo Scientific) wavelength range of 400–800 nm. Measurements were conducted in triplicates.

### Determination of viscosity

The viscosity of DES was carried out using a MCR102 Modular Compact Rheometer (Anton Parr) fitted with a parallel plate geometry with 50 mm of diameter (PP50, Anton Parr) and 1 mm of gap. Measurements of viscosity of the systems were performed in the temperature range of 60–20 °C (2 °C/min). Measurements were conducted in triplicates.

### Supercritical carbon dioxide extraction and dispersion in DES

The CO_2_ extraction was carried out in a lab-scale apparatus with the following main specifications: pneumatic pump (Williams P250V300), mass flow meter (Rheonik RHM 007), tubular extractor (316SS; 570 mm length, 24 mm I.D.; HiP), back-pressure regulator (Tescom Europe, model 26-1700, Selmsdorf, Germany), and separator (Swagelok 316L-HDF4-500). The extractions were performed by using 30 g of plant material under the following conditions: pressure 200 bar, temperature 40 °C, CO_2_ flow 20 g/min, and extraction time 3 h. The parameters of extraction were selected based on the previously conducted experimental work^23^.

After the extraction was completed, the extract (0.5 g ± 10%) was dispersed upon depressurization directly in the prepared DES (10 mL) and homogenized by mixing on a vortex for 30 s. The volume of 10 mL DES was selected after testing with 5, 10, and 15 mL based on visual evaluation (homogeneity) and the possibility of easy homogenisation after dispersion into DES. The volume of 10 mL demonstrated to be the most adequate for facilitated mixing with the extract and homogenisation. Moreover, the control extract was collected without dispersion into DES. Each experiment was performed in triplicate. The control and CO_2_ extracts dispersed into DES (DES-CO_2_) were analysed at the beginning (0 month), after 3 months, and after 6 months of storage in transparent containers at room temperature in the dark, with mixing for 20 s prior to each analysis. No visual changes in the samples were observed during storage.

### Headspace solid-phase microextraction (HS-SPME) and gas chromatography with mass spectrometry analysis (GC–MS)

The headspace volatiles were extracted by a manual SPME (solid phase microextraction) fibers with a layer of divinylbenzene/carboxene/polydimiethylsiloxane (DVB/CAR/PDMS) and poly-dimethylsiloxane/divinylbenzene (PDMS/DVB) from Supelco Co (Bellefonte, PA, USA). Both fibers were conditioned according to the manufacturer’s instructions. For HS-SPME, the sample (1.5 g) was placed in a 15 mL glass vial and hermetically sealed with PTFE/silicone septa. The closed vial was placed in a water bath at 60 °C during equilibration (15 min), and the extraction time of 45 min was applied for HS-SPME. After the sampling, the SPME fiber was withdrawn into the needle, removed from the vial, and inserted into the injector (250 °C) of the GC–MS (gas chromatography-mass spectrometry) for 6 min for thermal desorption directly to the GC column. Agilent 8890 gas chromatograph (Agilent Technologies, Palo Alto, CA, USA) coupled to mass spectrometer (series 5977E, Agilent Technologies, Palo Alto, CA, USA) was used for the analysis. The analysed components were separated on HP-5MS capillary column (30 m × 0.25 mm, 0.25 μm, Agilent Technologies, Palo Alto, CA, USA). The injector temperature was set at 250 °C with split mode of 1:50. Helium was used as a carrier gas in a constant flow regime of 1 mL/min. The GC temperature program was set at 70 °C for 2 min followed by the temperature ramp of 3 °C/min to 200 °C, and afterwards the temperature was maintained constant for 15 min. The separated components were analysed with a mass spectrometry (70 eV) with a scan mode and *m*/*z* range of 30–300. The injector and detector temperatures were 250 and 300 °C, respectively. Qualitative identifications of the compounds were performed using Wiley 9 (Wiley, New York, NY, USA) and NIST 17 (National Institute of Standards and Technology, Gaithersburg, MD, USA) mass spectral libraries as well as the literature data of retention indices calculated with C_9_–C_25_ alkanes. The HS-SPME/GC-MS analysis of each sample was performed in three replicates and the results are expressed as mean data.

### Chemometric pattern recognition methods

HCA was carried out based on the division approach and Ward’s algorithm on the raw data set. The results of the clustering were formed as a dendrogram. The Euclidean distances were used as a distance measure. In order to gain a much clearer overview of the similarities and dissimilarities among the samples, the PCA was applied. Moreover, it was based on the correlations and the raw data set. The SRD analysis was based on the ranking of samples in regard to the reference ranking. The reference ranking in this study was defined as a row average value. The validation of the SRD procedure was done based on the comparison of ranks by the random numbers (CRRN) procedure and the sevenfold cross-validation approach^48,49^. All the chemometric calculations were based on average values of the results obtained using two fibers during storage due to the similarities in terms of distribution and the trend of identified components. The detailed explanation of the applied chemometric methods can be found in the study by Miller and Miller^50^.

### Statistical analysis

All analyses were carried out in triplicate and the results were expressed as means ± standard deviation. Mean values were considered significantly different at a *p* < 0.05 confidence level, after the performance of the one-way ANOVA statistical analysis followed by Tukey’s HSD post hoc test.

## Conclusion

For the first time, this study investigated the approach which includes the attainment and stabilization of aroma volatile compounds by applying green solvents, supercritical CO_2_ and DESs. Supercritical CO_2_ extracts were dispersed into different DESs and their HS profiles were monitored during 6 months of storage. In the control CO_2_ extract, it was observed that there was a change in the HS profile and the formation of new components which can degrade the quality of the extract and limit its application. On the other hand, DESs demonstrated their capacity as stabilization media. Among the investigated DESs, Bet:EG demonstrated to be the most adequate system for maintaining stability, considering the very low number of newly formed components after 6 months of storage at room temperature.

The dispersion of the CO_2_ extracts into DES represents a green and simple solution that can adequately respond to the rising demand for volatile aroma components of natural origin while rationally utilizing natural resources. In addition, DESs-CO_2_ *L. stoechas* extracts have immense potential for application in health, cosmetic, perfume, aromatherapy, food, beverage, chemical, and insecticide-related areas due to the presence of numerous components with significant biological activity.

An industrial scale-up of this solution for component stabilization provides additional benefits: DESs provide 100% yield, while the CO_2_ can be recycled, hence there is no generation and accumulation of solvent waste. Moreover, considering that the costs of transportation and storage at low temperatures represent high expenses, preserving lipophilic compounds of *L. stoechas* in DES mixtures at room temperature represents an additional and significant advantage.

## Supplementary Information

Supplementary Figure S1.

## Data Availability

All data generated or analyzed during this study is included in article and its supplementary information files.
