# Supplementary material for: A new green approach for Lavandula stoechas aroma recovery and stabilization coupling supercritical CO2 and natural deep eutectic solvents
Source: Sci Rep. 2023 Aug 1;13:12443. doi: 10.1038/s41598-023-39516-5 (PMC10394027; doi:10.1038/s41598-023-39516-5)

**A new green approach for *Lavandula stoechas* aroma recovery and stabilization coupling supercritical CO_2_ and natural deep eutectic solvents**

**Jelena Vladić^1,2^, Strahinja Kovačević^2^, Silvia Rebocho^1^, Alexandre Paiva^1^, Stela Jokić^3^, Ana Rita Duarte^1*^, Igor Jerković^4,*^**

^1^ Faculdade de Ciências e Tecnologia, Universidade Nova de Lisboa, Caparica, 2829-516, Portugal

^2^ Faculty of Technology, University of Novi Sad, Novi Sad, 21000, Serbia

^3^ Faculty of Food Technology Osijek, University of Josip Juraj Strossmayer of Osijek, Osijek, 31000, Croatia

^4^ Faculty of Chemistry and Technology, University of Split, Split, 21000, Croatia

Corresponding authors: [igor@ktf-split.hr](mailto:igor@ktf-split.hr); [ard08968@fct.unl.pt](mailto:ard08968@fct.unl.pt)

**Supplementary data**

Fig. S1. Loadings plot of a) PC1-PC2 and b) PC1-PC3 space. The figures were created with TIBCO Software Inc. (2020). Data Science Workbench, version 14. http://tibco.com.

a)


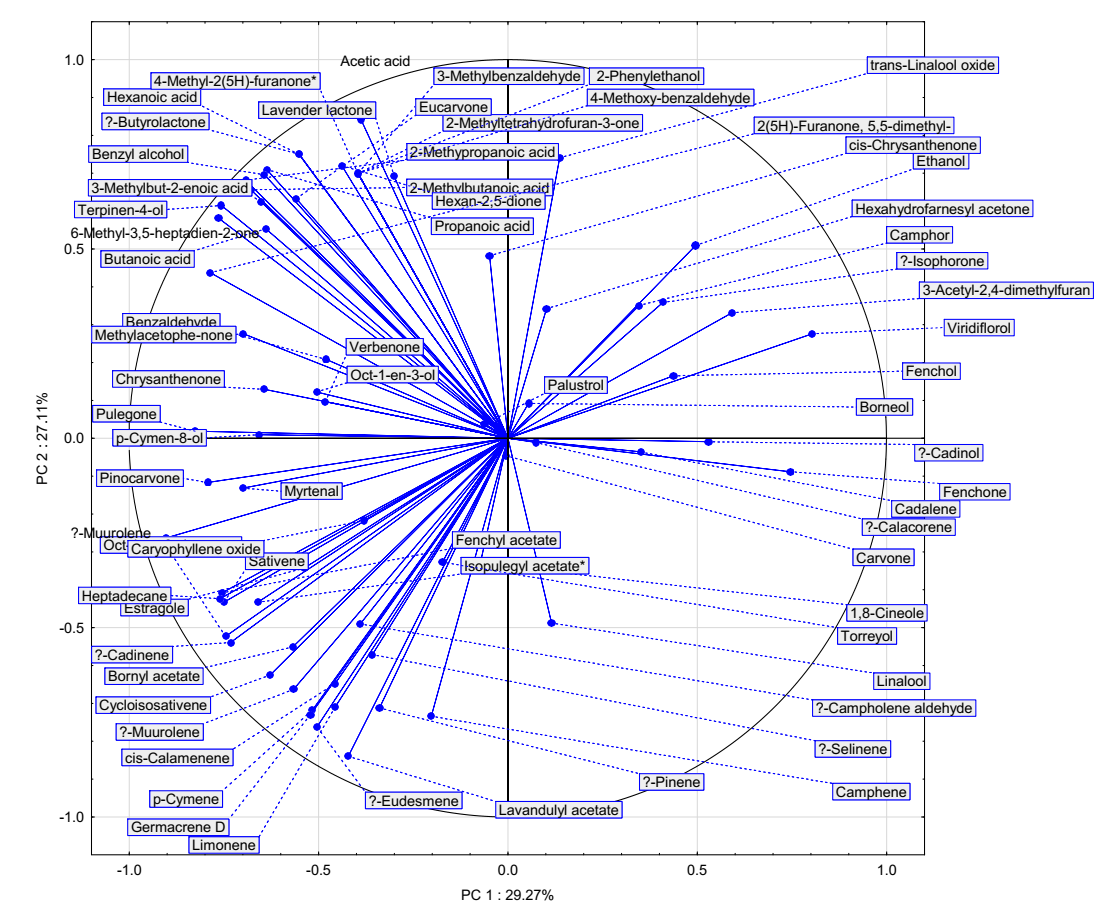


b)


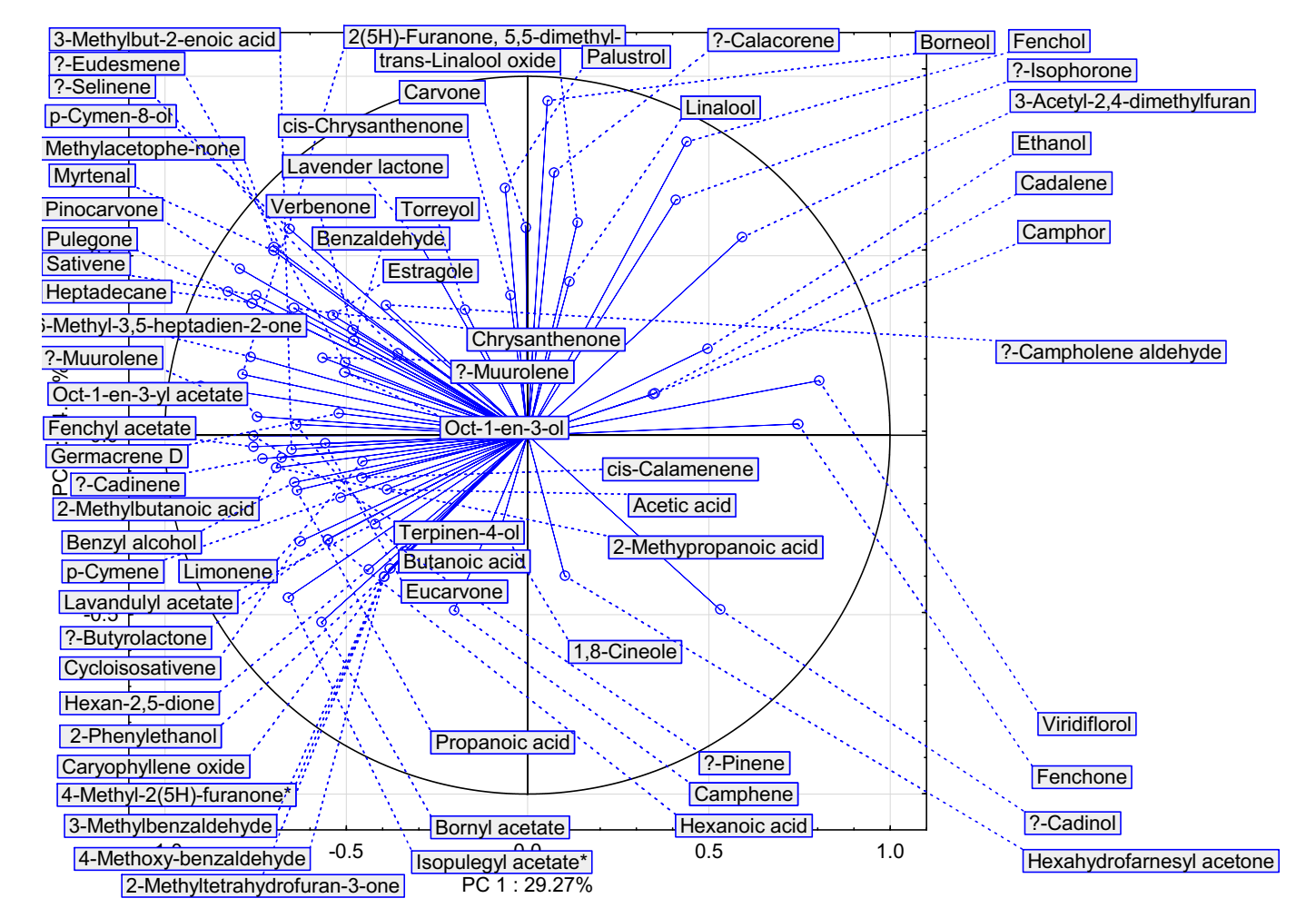

Supplement: Supplementary file 1 — Supplementary Figure S1. [file 41598_2023_39516_MOESM1_ESM.docx]
